# Ferroptosis in osteoporosis: core mechanisms, cell-specific effects in bone remodelling, and therapeutic perspectives

**DOI:** 10.3389/fendo.2026.1945454

**Published:** 2026-09-25

**Authors:** Zhihao Wu, Gaohai Shao

**Affiliations:** Department of Orthopaedics, The Affiliated Yongchuan Hospital of Chongqing Medical University, Chongqing, China

**Keywords:** bone marrow mesenchymal stem cells, bone remodelling, ferroptosis, osteoblasts, osteoclasts, osteocytes, osteoporosis

## Abstract

Osteoporosis is an endocrine and metabolic bone disease driven by insufficient bone formation, enhanced bone resorption, and disrupted coupling of bone remodelling. Ferroptosis, characterised by iron-dependent membrane phospholipid peroxidation and failure of anti-lipid-peroxidation defences, is emerging as a mechanistic link between oestrogen deficiency, glucolipotoxicity, glucocorticoid exposure, ageing, iron overload, and dysfunction of bone-remodelling cells. This structured narrative review summarises iron homeostasis, lipid peroxidation, the System Xc^−^–GSH–GPX4 axis, ferritinophagy, mitochondrial metabolism, and non-GPX4-dependent defence systems, and compares the direction and strength of ferroptotic effects in osteoblasts, osteocytes, osteoclasts, and bone marrow mesenchymal stem cells. Current evidence indicates that ferroptosis in osteoblasts and bone marrow mesenchymal stem cells impairs osteogenic differentiation, matrix formation, and mineralisation; ferroptosis in osteocytes may further disrupt RANKL-related remodelling communication; whereas ferroptosis in osteoclasts may limit bone degradation under high-resorption conditions. This review further integrates the Nrf2, NOX4, Wnt/β-catenin, DNMT/GPX4, HIF-1α/NCOA4, FtMt/PINK1/Parkin, and metabolic regulatory pathways, compares dominant cellular targets across osteoporosis subtypes, and evaluates iron chelation, inhibition of lipid peroxidation, metabolic regulation, extracellular vesicles, epigenetic restoration, and selective induction of osteoclast ferroptosis. Overall, the therapeutic objective should not be uniform inhibition or induction of ferroptosis, but selective intervention according to cell identity, disease subtype, and bone-turnover status. Because the evidence remains predominantly preclinical, translation will require cell-specific models, standardised criteria for defining ferroptosis, and bone-targeted delivery.

## 1 Introduction

Osteoporosis is an endocrine and metabolic bone disease characterised by reduced bone mass, deterioration of bone microarchitecture, and decreased bone strength. Its development depends not only on enhanced osteoclast-mediated bone resorption, but also on dysfunction of osteoblasts, osteocytes, and bone marrow mesenchymal stem cells (BMSCs) and on impaired coupling of bone remodelling (1–7). Iron is essential for cellular metabolism and skeletal homeostasis; however, iron overload expands the catalytically active labile iron pool and promotes reactive oxygen species generation and lipid peroxidation, whereas iron deficiency can impair collagen synthesis, mitochondrial function, and osteogenic differentiation (8–11). Thus, disturbed iron homeostasis provides an important pathological background for metabolic stress and ferroptosis in osteoporosis (9, 11–14).

Ferroptosis is a regulated cell-death programme driven by iron-dependent membrane phospholipid peroxidation (15, 16). In bone-forming cells, catalytic iron and ROS can initiate oxidation of PUFA-containing membrane phospholipids; when propagation of this damage overwhelms the System Xc^−^–GSH–GPX4 defence and other protective systems, membrane integrity and cell viability can be lost (8, 17–21). Impaired differentiation and mineralisation are functional consequences of this injury, but do not themselves identify its mode of cell death. Susceptibility therefore reflects the balance among iron burden, oxidisable membrane lipids, and anti-lipid-peroxidation systems rather than any single marker (8, 17, 18, 20–25).

Ferroptosis complements rather than replaces established mechanisms involving oestrogen deficiency, inflammation, RANKL-driven osteoclastogenesis, glucocorticoid injury, and ageing-related oxidative stress (26–32). They are introduced here because iron dysregulation and failure of lipid-peroxide detoxification provide a mechanistic connection between these established causes of osteoporosis and ferroptotic injury in defined bone-cell models (8, 15–18, 20, 21, 25, 33, 34). Importantly, increased reactive oxygen species, malondialdehyde, or mitochondrial damage alone does not establish ferroptosis; iron dependence, membrane phospholipid peroxidation, and ferroptosis-specific rescue should be demonstrated collectively (15, 16, 35–37).

The role of ferroptosis in osteoporosis is cell-type dependent (8, 17, 18, 20, 21, 25, 34, 38). It generally impairs osteogenic differentiation and matrix formation in osteoblasts and BMSCs, disrupts osteocyte viability and RANKL/OPG-related remodelling communication, but may restrict bone resorption when selectively induced in pathologically overactive osteoclasts (8, 17, 18, 20, 21, 25, 34, 38). Its net skeletal effect must therefore be interpreted according to cell identity, disease subtype, and bone-turnover status.

This review integrates the core mechanisms of ferroptosis with evidence from osteoblasts, osteocytes, osteoclasts, and BMSCs; examines major signalling pathways across osteoporosis subtypes; and evaluates therapeutic strategies and translational barriers. Its central framework links mechanism, cell type, disease context, and therapeutic direction while distinguishing direct mechanistic evidence from preliminary or extrapolated findings. The abbreviation BMSCs denotes bone marrow mesenchymal stem cells throughout this review, following the terminology of the cited studies; it does not imply that every cultured stromal population has demonstrated *in vivo* self-renewal and multipotency. Where stemness has not been established, “mesenchymal stromal cells” is the more precise biological designation (39).

## 2 Literature search and evidence assessment

This article is a structured narrative review, rather than a systematic or scoping review. PubMed/MEDLINE and Web of Science Core Collection were searched for English-language publications available up to 06 September 2026; the searches were re-run on that date during revision, and database-displayed counts were recorded before deduplication or screening. The reference lists and citing articles of key original studies were also screened to identify additional evidence. Search terms included combinations of “ferroptosis”, “osteoporosis”, “bone remodelling”, “osteoblast”, “osteocyte”, “osteoclast”, “bone marrow mesenchymal stem cell”, “iron homeostasis”, “GPX4”, “ferritinophagy”, “mitophagy”, “postmenopausal”, “diabetes”, “glucocorticoid”, “ageing”, and “therapy”. The terms “selenium” and “selenoprotein” were additionally used when assessing the structure and function of GPX4. Complete database-specific Boolean strings, the actual execution date, and database-displayed record counts are provided in **Supplementary Table S1**.

Priority was given to seminal original studies defining the core mechanisms of ferroptosis, studies directly examining bone-remodelling cells or animal models of osteoporosis, and investigations that provided causal evidence through iron chelators, ferrostatin-1, liproxstatin-1, GPX4/SLC7A11 modulation, or genetic intervention. For interpretation, bone-cell and osteoporosis-model original studies provide disease-specific evidence, whereas non-bone original studies provide biochemical background only. Reviews may guide citation tracing but do not constitute independent experimental validation; conference abstracts without full reports and non-English publications fall outside the stated full-text English-language evidence scope. The exported results from the bone-cell, subtype, and intervention searches were checked at title level, after which abstracts and, where necessary, full texts were assessed for potentially conclusion-changing bone studies. The broad core-mechanism search was used for biochemical background and citation tracing rather than as an exhaustive eligibility set; database export limits therefore did not define a fixed study corpus. Studies reporting only ROS, malondialdehyde, mitochondrial injury, or reduced viability are classified as oxidative injury unless additional evidence specifically links that injury to ferroptosis. Because this was a mechanism-oriented narrative review, no quantitative meta-analysis was performed. A PRISMA-style flow diagram is not presented because this synthesis was not designed as exhaustive systematic screening of a fixed study corpus. Providing a retrospective flow diagram without verifiable systematic-screening records would imply a selection process not documented here.

To avoid interpreting different levels of evidence as equivalent, a three-tier evidence framework was used (**Table 1**). Evidence levels were assigned to specific claims rather than entire publications; consequently, one study could support different levels for different conclusions. When findings conflicted, greater weight was given to ferroptosis-specific rescue, genetic intervention, and *in vivo* skeletal phenotypes rather than to the simple number of studies. This framework was used to calibrate the strength of conclusions in this narrative review and was not intended to replace GRADE assessment in a systematic review.

**Table 1:** Three-tier evidence assessment framework and its application in this review.

Level	Minimum evidence requirements	Permitted conclusion	Recommended wording
Level A	Iron-dependent membrane phospholipid peroxidation demonstrated in a defined bone-related cell population, together with ferroptosis-specific rescue, genetic validation, and an *in vivo* skeletal phenotype.	Supports a causal contribution to a bone-remodelling outcome and may justify consideration as a preclinical intervention target.	“Demonstrates”, “drives”, or “mediates”; conclusions remain restricted to validated models.
Level B	Direct cellular mechanism and specific rescue, with partial *in vivo* evidence or a defined outcome in bone formation, bone resorption, or bone microarchitecture, but without a complete genetic-to-*in vivo* causal chain.	Supports involvement in the disease process or therapeutic potential, but cannot be extrapolated as a confirmed mechanism in humans.	“Supports”, “participates in”, “may constitute”, or “has potential”.
Level C	Correlational observation, general oxidative-stress indices, extrapolation from non-skeletal systems, or a bone-cell model lacking iron dependence and ferroptosis-specific rescue.	Used only to propose a candidate mechanism, interpretive framework, or hypothesis. Evidence from a single model may still meet Level A or B criteria but remains limited in external replication.	“Suggests”, “may”, “is hypothesised”, or “requires validation”.

## 3 Core mechanisms of ferroptosis

Ferroptosis is closely integrated with cellular metabolism because multiple metabolic pathways can generate reactive oxygen species and lipid peroxides (22, 33, 40). Amino acid metabolism, glucose metabolism, iron metabolism, mitochondrial respiration, autophagy, and NADPH-dependent redox reactions can all modify ferroptosis susceptibility (33, 40–43). In bone tissue, these processes can be organised into three interconnected layers: iron provides catalytic oxidative pressure, PUFA-containing phospholipids provide oxidisable membrane substrates, and GSH, GPX4, and other radical-trapping systems provide defence capacity (22, 24, 44–47). Because the four major bone-remodelling cell populations differ in their dependence on energy metabolism, iron homeostasis, and redox signalling, the same ferroptotic pressure can lead to different remodelling outcomes (4–7, 17, 18, 20, 31, 34, 48).

### 3.1 Iron metabolism and iron overload

Disrupted iron metabolism is closely associated with osteoporosis, and both iron deficiency and iron overload can interfere with bone remodelling (9, 11, 13, 14, 49). Iron is required for oxygen transport, mitochondrial respiration, enzymatic catalysis, collagen synthesis, and mineralisation, and also influences osteoblast differentiation and osteoclast activity (9–12, 50). Accordingly, iron should be regarded as a bidirectional regulator of skeletal homeostasis rather than solely as a toxic factor.

Iron restriction impairs skeletal microarchitecture and reduces mineralisation in cultured hFOB osteoblasts (11). These findings support impaired bone formation, but do not establish a uniform increase in bone resorption. Indeed, severe iron deficiency reduced both bone formation and bone resorption in a rat study, indicating that the remodelling response depends on the severity and experimental context of deficiency (51). These effects are distinct from iron-catalysed lipid peroxidation during iron overload.

Iron overload expands the labile iron pool and enhances Fenton chemistry, thereby increasing hydroxyl radicals, oxidative stress, mitochondrial damage, and lipid peroxidation (9, 10, 18, 52). Experimental models show trabecular loss, cortical thinning, increased resorption, and deterioration of bone microarchitecture (9, 10, 12, 49, 52, 53). Clinical studies likewise associate hereditary haemochromatosis or elevated ferritin with osteoporosis and accelerated bone loss (13, 14).

Osteoblasts appear particularly sensitive to excess iron. Increased intracellular iron reduces mitochondrial membrane potential, increases ROS production, and can trigger regulated cell death (10, 52). Although early studies emphasised apoptosis or general oxidative injury, later work demonstrated that iron-dependent lipid peroxidation and ferroptosis also contribute to osteoblast dysfunction (18).

Mechanistically, excess iron initiates membrane lipid peroxidation through the Fenton reaction, aggravates injury when System Xc^−^–GSH–GPX4 defence is insufficient, and disrupts mitochondrial iron handling and quality control. NCOA4-mediated ferritinophagy can further release ferritin-bound iron under specific stress conditions; however, iron-replete conditions may also promote HERC2-dependent NCOA4 degradation and limit ferritinophagy (8, 9, 16–18, 33, 41, 42, 52, 54, 55). These processes connect abnormal iron storage with ferroptotic stress rather than representing isolated oxidative events.

The effects of disturbed iron homeostasis on bone remodelling differ among cell types. Iron overload can impair differentiation and mineralisation in osteoblasts and BMSCs; in models with direct ferroptosis evidence, iron-dependent cell injury also compromises osteoblast or osteocyte viability and remodelling communication (8, 12, 17, 18, 20, 21, 25). Osteoclasts require iron for differentiation and resorption, whereas RANKL-associated NCOA4-mediated ferritinophagy can increase intracellular iron release and susceptibility to ferroptosis (31, 34, 50). The latter finding concerns a defined differentiation model and does not establish that iron overload invariably kills mature osteoclasts by ferroptosis.

Circulating Fe³^+^ is carried mainly by transferrin and internalised through TFR1-mediated endocytosis (40). In endosomes, STEAP proteins reduce Fe³^+^ to Fe²^+^, which DMT1/SLC11A2 exports to the cytoplasm to form the labile iron pool (40, 56, 57). ZIP14/SLC39A14 and ZIP8/SLC39A8 can mediate uptake of non-transferrin-bound iron (58, 59). The resulting catalytic iron fuels Fenton chemistry, whereas LOXs and POR-dependent electron transfer can promote enzymatic phospholipid peroxidation, thereby influencing ferroptosis susceptibility (18, 52, 60, 61) (**Figure 1**). Fe²^+^ and Fe³^+^ denote ferrous and ferric iron, respectively; “Fe” without an oxidation state refers to iron generically.

**Figure 1:**
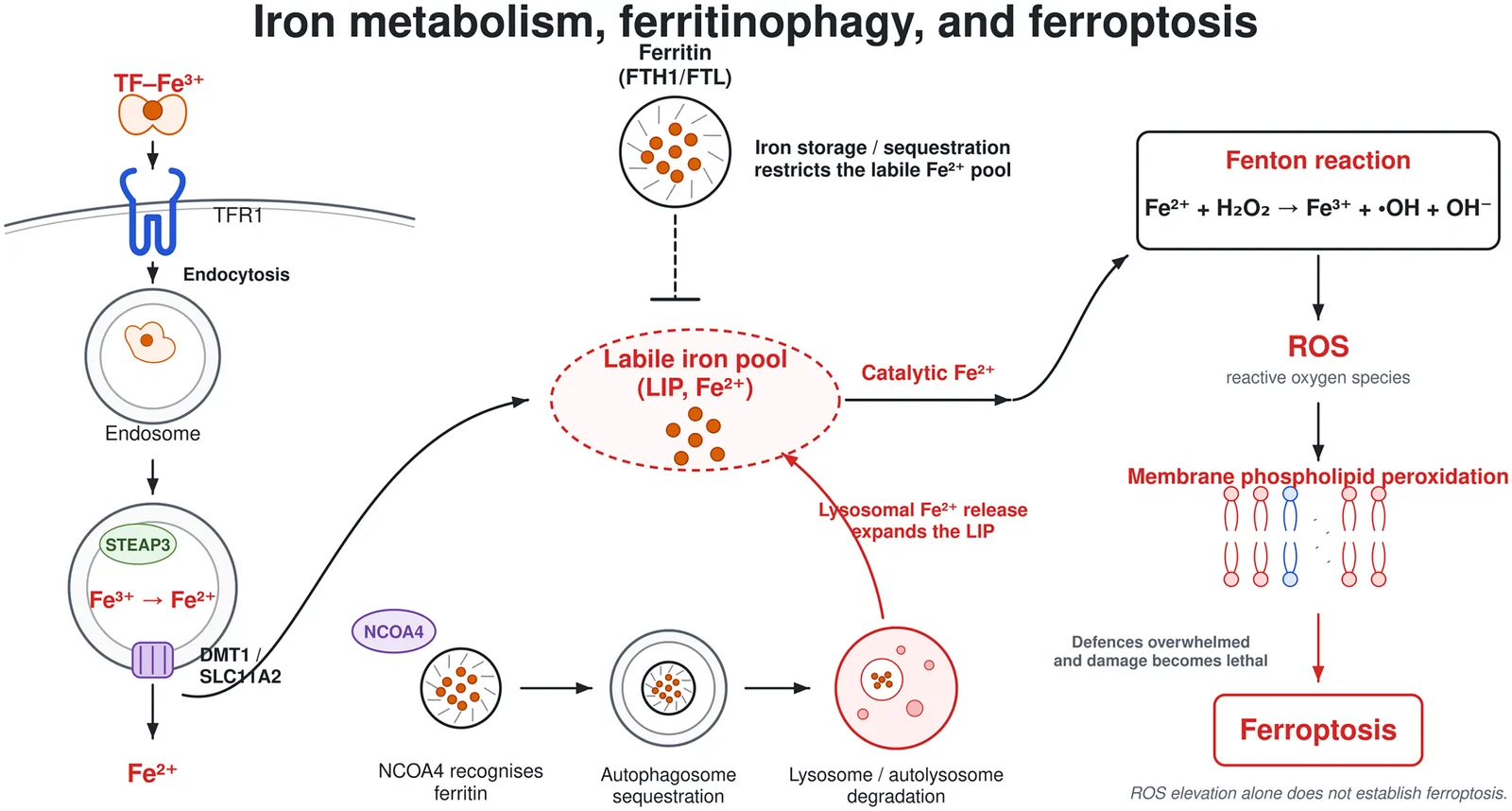
Iron metabolism, ferritinophagy, and susceptibility to ferroptosis. The final arrow denotes lethal iron-dependent phospholipid peroxidation when protective capacity is insufficient; elevated ROS alone does not establish ferroptosis.

### 3.2 Lipid peroxidation and reactive oxygen species accumulation

Lipid peroxidation is the direct execution step of ferroptosis (22, 23). Free polyunsaturated fatty acids (PUFAs) are readily oxidised, but their toxicity is generally fully manifested only after they are esterified into membrane phospholipids (22, 23). ACSL4 converts PUFAs such as arachidonic acid and adrenic acid into the corresponding fatty acyl-CoAs, and LPCAT3 further incorporates them into membrane phospholipids such as phosphatidylethanolamine, thereby providing substrates for subsequent peroxidation (22, 23). In contrast, monounsaturated fatty acids lack bis-allylic structures, are generally less susceptible to peroxidation, and can reduce cellular sensitivity to ferroptosis to some extent (62) (**Figure 2**).

**Figure 2:**
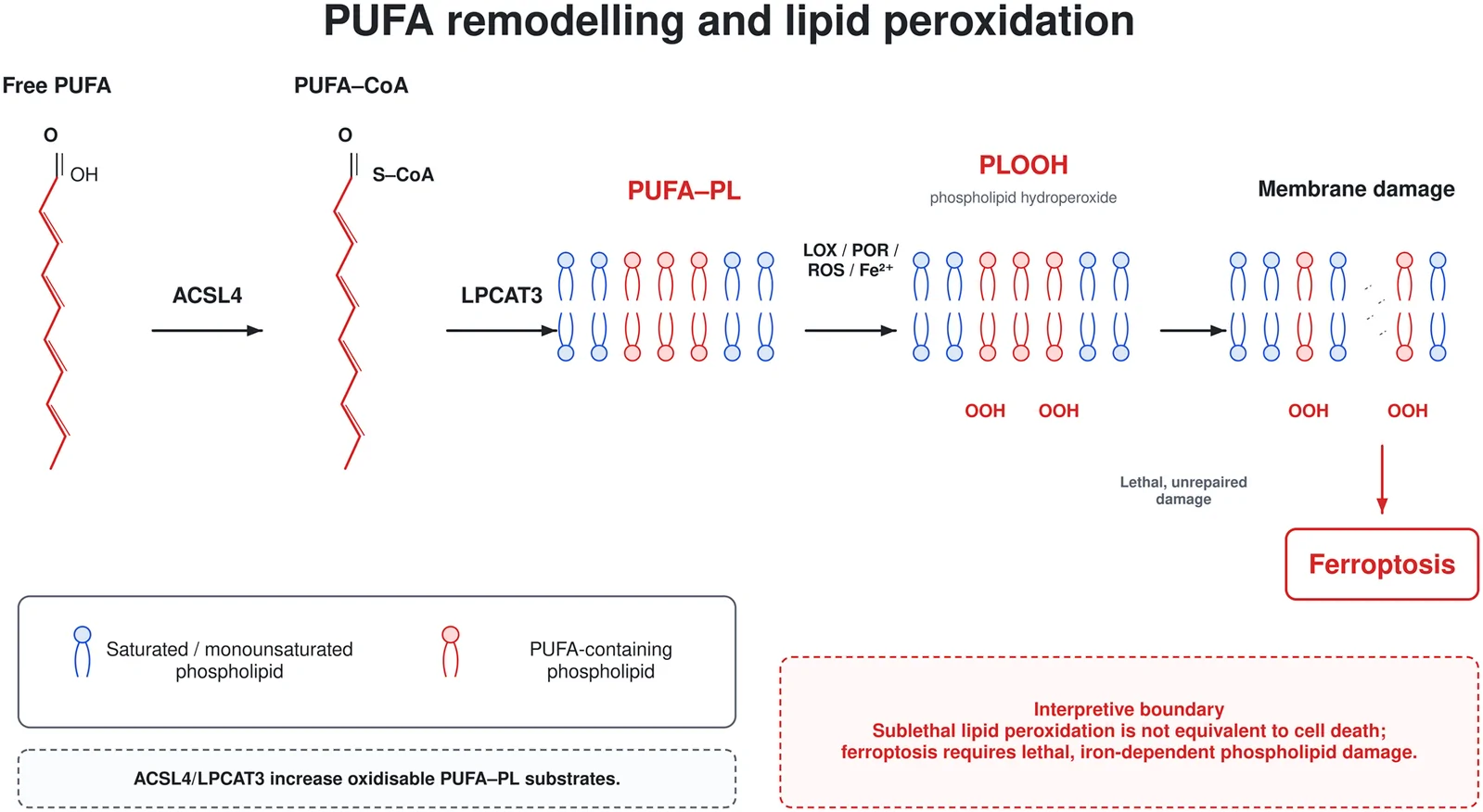
PUFA remodelling and lipid peroxidation during ferroptosis. Propagation of phospholipid oxidation must culminate in lethal membrane injury to constitute ferroptosis; sublethal lipid peroxidation is not equivalent to cell death.

Peroxidation of membrane PUFA-PLs can occur through enzymatic and nonenzymatic mechanisms (22, 23). Nonenzymatic reactions depend mainly on the iron-catalysed Fenton reaction, in which excess Fe²^+^ reacts with hydrogen peroxide to generate hydroxyl radicals and thereby initiates a lipid-radical chain reaction (9, 18, 52). Enzymatic lipid peroxidation involves lipoxygenases (LOXs), cytochrome P450 oxidoreductase (POR), and other membrane electron-transfer proteins (60, 61). Although the requirement for LOXs is not fully consistent across different models, their relationships with PUFA-PL oxidation, GPX4 function, and radical-trapping antioxidants indicate that ferroptosis is not a single-enzyme reaction, but rather the result of a combined imbalance in lipid substrates, iron catalysis, and antioxidant defence (22, 63).

In this process, ROS act both as initiators of lipid peroxidation and as amplifiers of injury (23, 33, 60). The mitochondrial respiratory chain, iron-catalysed reactions, POR, the NOX family, and inflammation-related oxidase systems can all increase ROS generation (33, 60). It should be noted that ferroptosis is not equivalent to general oxidative stress; ROS can initiate ferroptosis when they drive propagating oxidation of membrane PUFA-PLs that overwhelms protective systems and culminates in lethal membrane injury. This mechanistic sequence must be demonstrated in the model under discussion, rather than inferred from ROS elevation alone (15, 16, 23, 24).

When phospholipid hydroperoxides (PLOOHs) cannot be promptly reduced to the corresponding lipid alcohols, peroxyl-radical reactions continue to propagate and generate secondary products such as MDA and 4-HNE (23, 24, 60, 64). These products can react with proteins, nucleic acids, and membrane lipids, disrupt membrane integrity, and impair organelle function. The key feature of ferroptosis is not merely an increase in total ROS, but the accumulation of lipid peroxides within biological membranes beyond the clearance capacity of defence systems such as GPX4 (35–37). Membrane-repair responses can delay death despite lipid oxidation, further separating an upstream oxidative signal from execution of ferroptosis (65).

Lipid peroxidation has particular significance in bone tissue (8, 17, 18, 20, 21, 25, 34). Osteoblasts, osteocytes, and BMSCs all depend on stable mitochondrial function and membrane structure to maintain differentiation, mineralisation, and intercellular communication; once membrane lipids undergo sustained oxidation, the injury is rapidly translated into reduced osteogenic capacity or abnormal remodelling signals (8, 17, 18, 20, 21, 25). Osteoclasts require controlled ROS signalling for differentiation and resorptive activity, but this requirement does not establish a requirement for phospholipid peroxidation or ferroptosis. RANKL-dependent redox signalling and ferritinophagy-associated ferroptotic injury are distinct processes whose effects depend on differentiation stage and experimental conditions (31, 34). Across osteoporosis models, distinct stimuli—including iron overload, high glucose, glucocorticoids, inflammation, and ageing—may converge on lipid peroxidation, but ferroptosis should be concluded only when the model also demonstrates lethal iron-dependent membrane injury and appropriate rescue (8, 17, 18, 20, 21, 25, 66–68).

### 3.3 System Xc^−^–GSH–GPX4 axis

GPX4 is a selenoprotein that reduces toxic lipid hydroperoxides to less harmful lipid alcohols. It is therefore the most critical enzyme for preventing the accumulation of lipid hydroperoxides in most cells (16, 24, 64). GPX4 has multiple isoforms, including cytosolic, mitochondrial, and nuclear forms (16, 24). Although mitochondrial GPX4 may also help suppress ferroptosis under certain conditions, cytosolic GPX4 appears to be the most critical form for preventing ferroptosis. GPX4 catalytic activity requires GSH and a selenocysteine-containing active site, whereas selenium availability may influence GPX4 biosynthesis and anti-ferroptotic capacity (16, 69). GSH is synthesised from three amino acids—cysteine, glycine, and glutamate—and cysteine acquisition is the principal rate-limiting factor in this reaction; intracellular cysteine can be derived from cystine (70, 71). In mammals, cystine is imported mainly through the System Xc^−^ cystine/glutamate antiporter for subsequent GSH synthesis (70–72). System Xc^−^ is a heterodimer composed of a light chain (xCT, encoded by solute carrier family 7 member 11 [SLC7A11]) and a heavy chain (4F2, also known as CD98, encoded by solute carrier family 3 member 2 [SLC3A2]), with xCT determining the substrate specificity of System Xc^−^ (70). xCT is a specific amino acid transporter that imports cystine into the cell while exporting glutamate. The ferroptosis inducer RSL3 binds directly to GPX4 and inactivates it, whereas erastin indirectly inactivates GPX4 by targeting and blocking SLC7A11, thereby reducing cystine uptake (15, 16, 72). SLC7A11 activity is positively regulated by nuclear factor erythroid 2-related factor 2 (Nrf2) and can be negatively regulated by TP53 (73, 74) (**Figure 3**).

**Figure 3:**
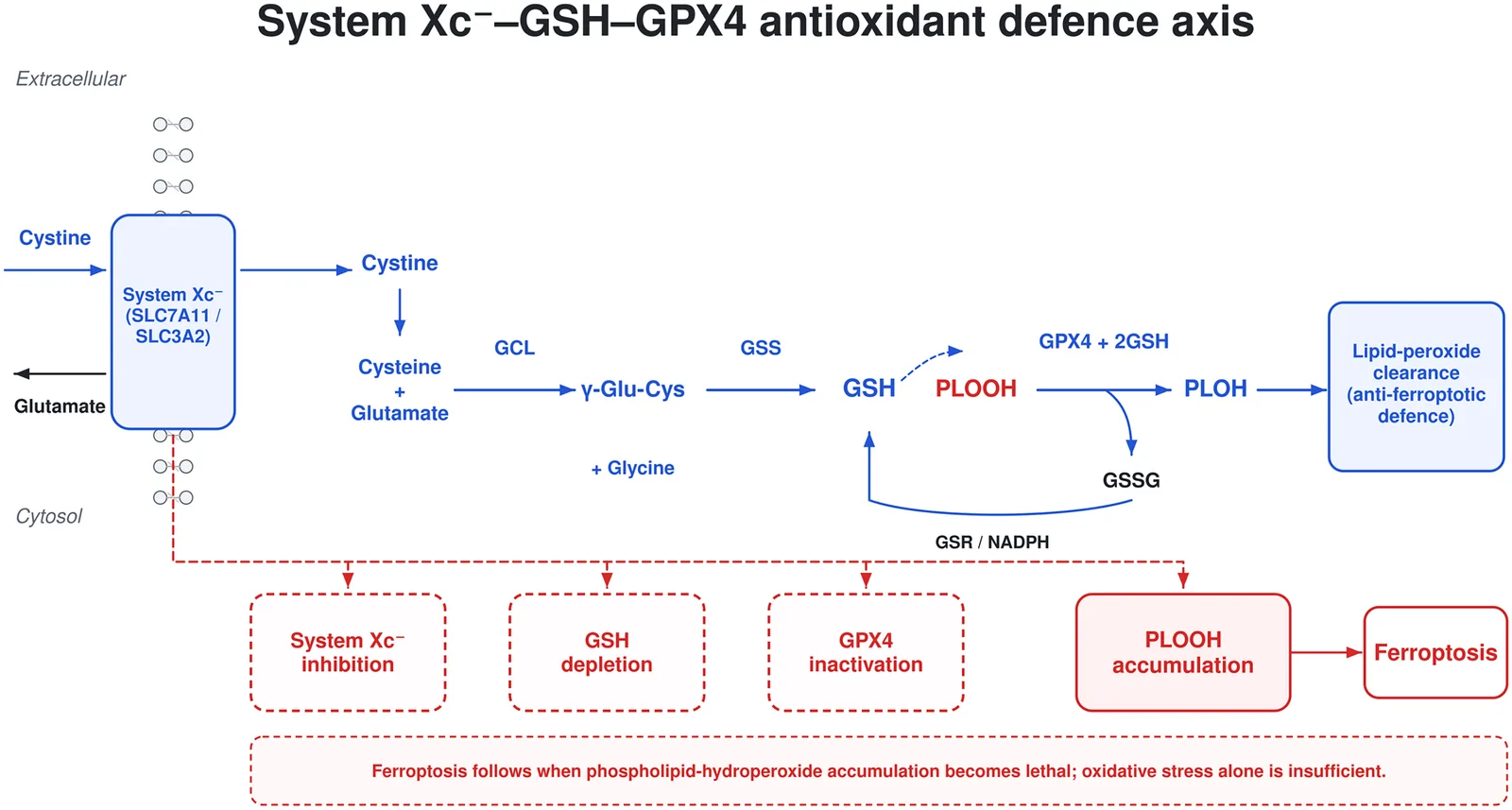
The system Xc^−^–GSH–GPX4 axis maintains resistance to ferroptosis.

GPX4 is the only member of the GPX family with phospholipid hydroperoxidase activity and can directly reduce phospholipid hydroperoxides (PLOOHs) to the corresponding phospholipid alcohols (PLOHs); this catalytic reaction requires GSH as a cofactor (16, 24, 64). First, PLOOH oxidises the selenol at the active site of GPX4 (GPX4-SeH), generating a selenenic acid intermediate (GPX4-SeOH) (24, 64). Second, GPX4-SeOH reacts with GSH to form a selenium-glutathione adduct (GPX4-Se-SG). Finally, through reaction with a second GSH molecule, GPX4-Se-SG is converted back to the selenol form (GPX4-SeH), with simultaneous release of oxidised glutathione (GSSG) (24, 64). Subsequently, NADPH is used to reduce GSSG back to GSH. Thus, GPX4 uses two GSH molecules to reduce a phospholipid hydroperoxide to its corresponding alcohol (16, 24, 64). In this mechanism, GPX4 clears lipid peroxides through its catalytic selenocysteine residue and two electrons supplied primarily by glutathione (GSH), although other small-molecule thiols and even protein thiols may also provide these electrons (24). In osteoporosis, impairment of the System Xc^−^–GSH–GPX4 axis reduces the ability of osteoblasts and osteocytes to clear phospholipid peroxides (8, 17, 18, 21, 75). This results in lipid-peroxide accumulation, impaired osteogenic differentiation, and a shift in bone remodelling towards bone loss (8, 17, 18, 21, 75). Recent evidence further indicates that, in osteoporosis, GPX4 may undergo epigenetic silencing through DNMT-mediated promoter methylation, suppressing GPX4 expression and thereby linking abnormal DNA methylation to osteoblast ferroptosis and bone loss (75).

The catalytic activity of GPX4 depends on its selenocysteine residue; however, selenium is considered here only as a structural and functional component of GPX4 rather than as an independent mechanistic theme in osteoporosis. Replacing selenocysteine in GPX4 with cysteine reduces its resistance to irreversible overoxidation, and selenium treatment can induce an adaptive anti-ferroptotic programme that includes GPX4 (24, 76). Nevertheless, causal evidence directly linking selenium status, GPX4 function in bone cells, and osteoporosis outcomes remains limited. Selenium deficiency can impair bone microarchitecture in mice, whereas a randomised trial in postmenopausal women did not show that short-term selenium supplementation improved bone-turnover markers (77, 78). Selenium status should therefore be treated as a potential modifier of GPX4 function rather than as an established anti-osteoporosis therapy.

### 3.4 Ferritinophagy and mitochondrial iron metabolism

Intracellular iron is stored primarily in ferritin, a heteropolymeric iron-storage protein composed of ferritin heavy chain 1 (FTH1) and ferritin light chain (FTL) (41, 55). FTH1 has ferroxidase activity and converts the more reactive ferrous iron (Fe²^+^) into ferric iron (Fe³^+^), thereby allowing iron to be safely stored within the ferritin core (41, 55). FTL contributes to formation and structural stability of the ferritin core and ensures efficient iron storage. By restricting the labile iron pool (LIP), ferritin reduces the availability of catalytic iron for the Fenton reaction and lipid peroxidation (41, 55). Therefore, ferritin is not merely an iron-storage protein, but also an important regulator of ferroptosis sensitivity.

Ferritinophagy is a selective autophagic process that degrades ferritin and releases stored iron into the cytoplasm (41, 42, 54). This process is mediated mainly by nuclear receptor coactivator 4 (NCOA4), which recognises ferritin and delivers it to autophagosomes for lysosomal degradation (41, 55). By expanding the LIP, NCOA4-mediated ferritinophagy increases the availability of catalytic iron for the Fenton reaction and lipid peroxidation, thereby increasing susceptibility to ferroptosis (41, 42, 54). Conversely, inhibition of NCOA4 can reduce ferritin degradation, restrict iron release, and suppress ferroptotic injury (41, 42, 54). In experimental ferroptosis models, NCOA4 knockdown has been shown to protect cells from erastin-induced ferroptosis, whereas NCOA4 overexpression alone increases vulnerability to ferroptosis (42, 54). These findings establish ferritinophagy as a core mechanism linking iron storage, iron release, and ferroptosis execution.

Ferritinophagy is particularly relevant in osteoporosis because bone-remodelling cells are highly dependent on iron homeostasis and redox balance. Excessive ferritinophagy may aggravate ferroptosis in osteoblast-lineage cells and BMSCs by expanding the LIP and promoting lipid peroxidation, whereas regulation of ferritinophagy in osteoclasts may affect osteoclast survival and bone resorption (20, 34, 79). Thus, ferritinophagy provides a mechanistic explanation for how intracellular iron stores are converted into a source of ferroptotic stress under pathological conditions.

Mitochondrial iron metabolism represents another important dimension of ferroptosis regulation (33). Mitoferrin-1 (SLC25A37) and mitoferrin-2 (SLC25A28) participate in mitochondrial iron uptake, while DMT1 has also been implicated in mitochondrial iron transport in non-bone experimental systems (8, 33, 80). Excess mitochondrial iron promotes mitochondrial ROS generation, disrupts mitochondrial membrane potential, and causes oxidative injury, thereby increasing cellular susceptibility to ferroptosis (8, 21). Morphologically, ferroptotic cells commonly exhibit shrunken mitochondria, increased membrane density, reduced or absent cristae, and rupture of the outer mitochondrial membrane (15, 33). However, whether these mitochondrial changes actively drive ferroptosis or merely reflect ferroptotic stress remains unresolved. Their relevance to bone cells is supported by the dependence of osteoblast differentiation and osteocyte survival on mitochondrial function and redox homeostasis, but the transporter-level evidence should not be assumed to be bone-cell specific (8, 21, 33).

Mitochondrial metabolism can also influence ferroptosis through pathways other than iron transport (33, 40). Glutamine metabolism, the tricarboxylic acid cycle, and the mitochondrial electron-transport chain can support cystine deprivation-induced ferroptosis by promoting mitochondrial ROS generation (33, 40). This mechanism indicates that ferroptosis is not merely a consequence of iron overload, but also depends on the metabolic state and mitochondrial activity of the cell. In osteoporosis, metabolic stresses such as diabetes, ageing, and glucocorticoid exposure may sensitise osteoblast-lineage cells to ferroptotic injury by disrupting mitochondrial metabolism, redox balance, and mitochondrial quality control (8, 21, 25, 66–68, 81).

Mitochondrial ferritin (FtMt) provides a bone-related example illustrating how mitochondrial iron handling regulates ferroptosis (8). FtMt is an iron-storage protein localised within mitochondria that can buffer excess mitochondrial iron and thereby limit the availability of ferrous iron for ROS generation and lipid peroxidation. In osteoblasts, FtMt appears to exert an anti-ferroptotic effect by reducing oxidative stress caused by excess Fe²^+^ (8). Under diabetic metabolic stress, FtMt deficiency may aggravate mitochondrial iron dysregulation, increase ROS accumulation, and promote osteoblast ferroptosis. This mechanism is particularly relevant to type 2 diabetic osteoporosis, in which a high-glucose environment, mitochondrial dysfunction, and iron-dependent oxidative injury act together to impair osteoblast viability and osteogenic function (8).

Mitophagy further links mitochondrial iron metabolism to ferroptosis susceptibility in bone cells. In principle, mitophagy is a mitochondrial quality-control mechanism intended to remove damaged mitochondria and prevent excessive ROS production (8). However, under pathological metabolic stress, dysregulated or excessive mitophagy may also amplify mitochondrial injury and disrupt iron homeostasis. The PINK1/Parkin pathway is one of the principal mechanisms regulating mitophagy. In diabetic osteoporosis, FtMt deficiency may activate ROS/PINK1/Parkin-mediated mitophagy, thereby promoting mitochondrial dysfunction and osteoblast ferroptosis (8). This indicates that mitochondrial iron storage, mitochondrial ROS, and mitophagy are not independent events; rather, they form a regulatory axis that determines whether osteoblasts maintain mitochondrial homeostasis or progress towards ferroptotic injury.

Ferritinophagy also contributes to dysfunction of osteogenic progenitor cells (20). BMSCs are a major source of osteoblast-lineage cells, and their survival and osteogenic differentiation are essential for bone formation (4–7). When NCOA4-mediated ferritinophagy is excessively activated, ferritin degradation expands the LIP and promotes iron-dependent lipid peroxidation (41, 42, 54). In BMSCs, this process may reduce osteogenic differentiation capacity and contribute to bone loss. For example, pathological stimuli such as tobacco toxins have reportedly induced BMSC ferroptosis through NCOA4-mediated ferritinophagy, leading to reduced bone mass and deterioration of bone microarchitecture (20). Therefore, NCOA4-mediated ferritinophagy may be an upstream mechanism through which environmental or metabolic stress damages the osteogenic cell pool and promotes osteoporosis (20).

In glucocorticoid-induced osteoporosis, ferritinophagy-dependent ferroptosis also appears to be an important mechanism of osteoblast-lineage injury. Glucocorticoids can suppress osteogenic differentiation and increase oxidative stress in osteoblast-lineage cells (27, 28). Vascular endothelial cell-derived exosomes were reported to reverse glucocorticoid-induced inhibition of osteogenesis by suppressing ferritinophagy-dependent ferroptosis in osteoblasts (79). A separate endothelial progenitor cell-derived extracellular-vesicle study reported improved skeletal, osteogenic, and redox-related outcomes, but did not fully establish ferroptosis or a ferritinophagy-dependent mechanism (82). Thus, the ferritinophagy conclusion is supported by the former study, whereas the latter provides complementary but less mechanism-specific evidence.

The role of ferritinophagy in osteoclasts differs from that in osteoblasts and BMSCs. During RANKL-induced osteoclast differentiation, ferritinophagy may increase the release of ferritin-stored iron, thereby expanding the LIP and enhancing osteoclast susceptibility to ferroptosis. Hypoxia/HIF-1α signalling can counteract this process by suppressing ferritinophagy and reducing iron release and ROS accumulation, thereby protecting osteoclasts from ferroptosis. From the perspective of osteoporosis, this mechanism has dual implications (34). On the one hand, HIF-1α-mediated protection from ferroptosis may support osteoclast survival and bone resorption. On the other hand, pharmacological inhibition of HIF-1α or ferritin-related protective mechanisms may promote osteoclast ferroptosis and thereby reduce excessive bone resorption (34). Thus, ferritinophagy in osteoclasts may represent a potential antiresorptive target, whereas excessive ferritinophagy in osteoblasts and BMSCs is more likely to be a harmful process that should be suppressed.

Nevertheless, the role of ferritinophagy in osteoporosis should be interpreted cautiously. Current evidence indicates that ferritinophagy is not invariably harmful or protective in bone remodelling, but instead depends on the affected cell type and pathological environment (20, 34, 79). Most current evidence is still derived from cellular and animal models (20, 34, 79). Further studies are required to determine whether ferritinophagy-targeted interventions can achieve sufficient cell-type specificity, long-term safety, and therapeutic efficacy in the clinical treatment of osteoporosis.

### 3.5 Non-GPX4-dependent ferroptosis defence systems

The System Xc^−^–GSH–GPX4 axis is generally considered the central defence system against ferroptosis. However, ferroptosis cannot be fully explained by GPX4 activity alone (16, 44–47). Several non-GPX4-dependent antioxidant systems have now been identified, including the FSP1-CoQ10 system, the DHODH-CoQ10 system, and the GCH1-BH4 system. These pathways suppress ferroptosis primarily by trapping lipid peroxyl radicals, maintaining membrane redox balance, or limiting the accumulation of toxic phospholipid hydroperoxides (44–47). Although their functions have been studied most extensively in cancer and other nonskeletal disease models, they may also provide complementary protection against ferroptotic injury in bone cells.

Ferroptosis suppressor protein 1 (FSP1) represents one of the major non-GPX4-dependent anti-ferroptosis systems (44, 45). FSP1 is recruited to the plasma membrane through N-myristoylation and functions as an oxidoreductase, using NAD(P)H to reduce coenzyme Q10 (CoQ10) to ubiquinol (CoQH_2_). CoQH_2_ acts as a lipophilic radical-trapping antioxidant and suppresses ferroptosis by scavenging lipid peroxyl radicals within biological membranes. Thus, the FSP1-CoQ10 system can protect cells from lipid peroxidation even when GPX4 activity is impaired (44, 45). In the context of osteoporosis, this pathway is theoretically relevant because osteoblasts, osteocytes, and BMSCs are all susceptible to oxidative stress and lipid-peroxide accumulation under conditions such as iron overload, diabetic stress, glucocorticoid exposure, and ageing. However, direct evidence defining the function of FSP1 in bone-cell ferroptosis remains limited.

The mitochondrial enzyme dihydroorotate dehydrogenase (DHODH) provides another non-GPX4-dependent ferroptosis defence mechanism. DHODH is located in the inner mitochondrial membrane and participates in pyrimidine biosynthesis (47). More importantly in the context of ferroptosis, DHODH can reduce CoQ to CoQH_2_ within mitochondria, thereby suppressing mitochondrial lipid peroxidation. This mechanism is particularly relevant in cells with low or insufficient mitochondrial GPX4 activity (47). Because mitochondrial dysfunction, mitochondrial ROS accumulation, and impaired mitochondrial quality control are closely associated with osteoblast dysfunction and diabetic or age-related osteoporosis, the DHODH-CoQ10 axis may represent a potential mitochondrial anti-ferroptosis defence system in bone-forming cells. Nevertheless, whether DHODH directly regulates ferroptosis in osteoblasts, osteocytes, osteoclasts, or BMSCs in osteoporosis remains to be clarified.

The GCH1-BH4 system is another important ferroptosis defence pathway. GTP cyclohydrolase 1 (GCH1) catalyses the rate-limiting step in tetrahydrobiopterin (BH4) biosynthesis (46). BH4 functions as an endogenous antioxidant with radical-trapping capacity and can protect membrane lipids from peroxidative injury. In addition, GCH1-dependent BH4 production may alter membrane lipid composition by preserving phospholipids containing polyunsaturated fatty acyl chains, thereby reducing ferroptosis susceptibility (46). This system may be relevant to osteoporosis because ferroptosis in bone cells is driven by lipid peroxidation, and preservation of membrane lipid homeostasis may help maintain osteoblast and osteocyte viability. However, compared with GPX4, Nrf2/HO-1, NOX4, and ferritinophagy-related mechanisms, the role of GCH1-BH4 signalling in osteoporosis remains largely unexplored.

NADPH is a common metabolic requirement for several anti-ferroptosis systems. It supports glutathione reduction and the regeneration of reduced CoQ, vitamin K, and BH4, thereby contributing to cellular resistance to lipid peroxidation (44–46, 83). This protective role is not universal, because NADPH can also supply pro-oxidant enzymes. NADPH oxidases and NADPH-cytochrome P450 oxidoreductase (POR) can promote ROS generation and phospholipid peroxidation, whereas NADH-cytochrome b5 reductase (CYB5R1) uses NADH rather than NADPH (18, 60, 84). Accordingly, NADPH metabolism is best interpreted as a redox-balancing system whose effect depends on the enzymes receiving reducing equivalents, rather than as an exclusively protective factor.

Vitamin K- and vitamin E-related antioxidant mechanisms may also intersect with ferroptosis defence (83). FSP1 has been reported to reduce vitamin K to its hydroquinone form, which can act as a lipophilic antioxidant and inhibit lipid peroxidation (83). Similarly, α-tocopherol acts as a chain-breaking antioxidant and can interrupt the propagation of lipid radicals (36). These mechanisms are conceptually relevant to osteoporosis because oxidative stress and lipid peroxidation cause bone-cell injury. However, their direct anti-ferroptotic effects in osteoporosis remain insufficiently defined, and most available evidence continues to support broader antioxidant functions rather than a clearly established bone-specific ferroptosis mechanism.

## 4 Ferroptosis and bone remodelling

The preceding section described the biochemical foundations of ferroptosis in terms of iron burden, membrane lipid substrates, and antioxidant defence. The focus now shifts to bone-remodelling cells: ferroptosis in osteoblasts and bone marrow mesenchymal stem cells primarily reduces bone-forming capacity; ferroptosis in osteocytes can convert intracellular injury into abnormal RANKL-related remodelling communication; and ferroptosis in osteoclasts may limit bone degradation in high-resorption states (17, 18, 20, 21, 25, 34, 38). This cell-specific framework can be summarised as protection of the bone-forming compartment and potential selective induction of ferroptosis in the pathological bone-resorbing compartment (**Figure 4**).

**Figure 4:**
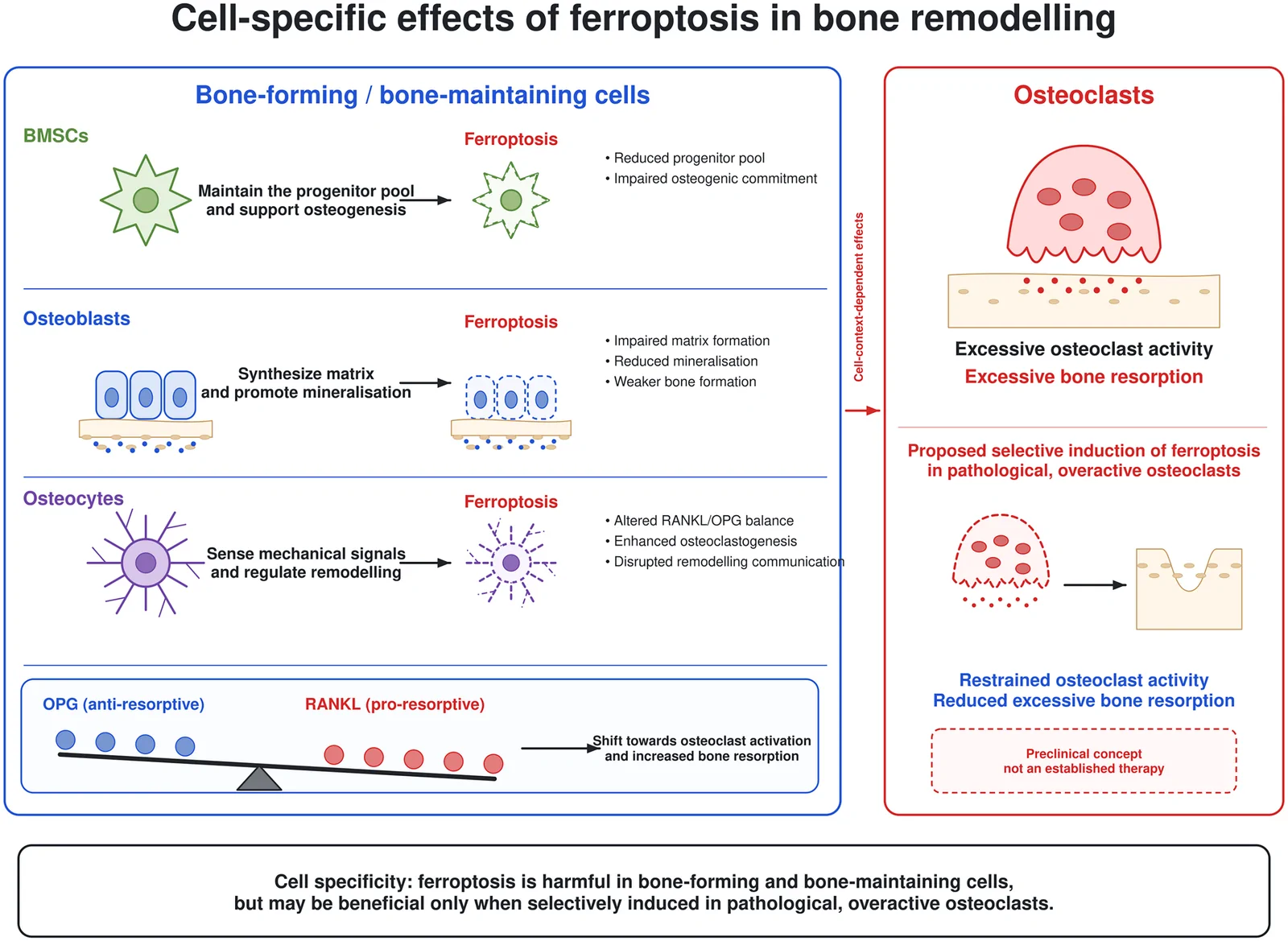
Cell-specific effects of ferroptosis in bone remodelling. Cell colours are consistent across **Figures 4**–**6**: osteoblasts, blue; osteocytes, purple; BMSCs, green; osteoclasts, red. Selective induction of osteoclast ferroptosis is a proposed antiresorptive strategy supported by preclinical findings, not an established clinical therapeutic approach.

### 4.1 Osteoblast ferroptosis and impaired bone formation

Osteoblasts are the direct effector cells of bone formation and are responsible for extracellular matrix synthesis, collagen deposition, alkaline phosphatase activity, and mineralised nodule formation (48, 85, 86). Direct experimental evidence of osteoblast ferroptosis has been reported in iron-overload models, diabetic high-glucose or high-glucose/high-lipid models, glucocorticoid-exposure models, and ageing-related models, with different degrees of mechanistic validation (8, 18, 19, 66–68, 75, 79). These contexts should be distinguished from inflammation-associated oxidative injury, which cannot be classified as ferroptosis solely from increased ROS. When lethal membrane lipid peroxidation is demonstrated together with iron dependence and appropriate rescue, osteoblast death can be attributed to ferroptosis.

The central consequence of osteoblast ferroptosis is not simply a reduction in cell number, but suppression of the osteogenic programme (18, 75). Iron overload can expand the labile iron pool and promote the Fenton reaction, while enzymatic ROS sources such as NOX4 can further amplify oxidative pressure, ultimately leading to increased lipid ROS, MDA, and 4-HNE, GSH depletion, and weakened SLC7A11- and GPX4-mediated defences (18). Functionally, these changes are manifested by reduced expression of osteogenic markers such as RUNX2, ALP, OCN, and COL1A1, decreased mineralised nodule formation, and impaired bone-forming capacity (18).

Multiple pathways convert this biochemical injury into osteogenic failure. The NOX4/ROS axis can convert iron overload into a sustained lipid-peroxidation signal; suppression of Wnt/β-catenin weakens the osteogenic transcriptional programme; and DNMT-mediated methylation of the GPX4 promoter suggests that osteoblast ferroptosis may also be regulated by stable epigenetic mechanisms rather than solely by acute oxidative injury (18, 75, 87). In diabetic osteoporosis, high-glucose-induced osteoblast ferroptosis can be partially reversed by interventions related to Nrf2/HO-1 or Nrf2/SLC7A11/GPX4, further indicating that anti-lipid-peroxidation capacity is closely linked to osteogenic function (19, 67).

From the perspective of disease context, iron overload-related osteoporosis places greater emphasis on expansion of the labile iron pool and amplification by NOX4; diabetic osteoporosis places greater emphasis on mitochondrial injury and reduced antioxidant defence induced by high glucose/high lipid conditions; and glucocorticoid- or ageing-related osteoporosis often involves simultaneous impairment of osteoblast survival and differentiation (8, 18, 19, 25, 66–68, 79). Although the upstream causes are not identical, they can converge on lipid peroxidation, insufficient GPX4 defence, and attenuation of osteogenic signalling (8, 18, 19, 25, 66–68, 75, 79).

Across these disease-specific models, experimentally validated ferroptotic injury in osteoblasts can reduce matrix production and mineralisation, thereby diminishing bone formation (8, 18, 19, 66–68, 75, 79). A direct effect of osteoblast ferroptosis on osteocyte–osteoclast communication, however, requires separate co-culture or lineage-specific validation. Interpretation should therefore remain tied to the pathological trigger, affected cell population, demonstrated death mechanism, and resulting skeletal phenotype.

### 4.2 Osteocyte ferroptosis and osteoclastogenic signalling

Osteocytes are embedded within the mineralised bone matrix and serve as central coordinators of bone remodelling. They regulate bone homeostasis through mechanosensation, mineral metabolism, and paracrine communication with osteoblasts and osteoclasts (1–3, 88). Therefore, the effects of osteocyte ferroptosis are not limited to a reduction in osteocyte number; more importantly, ferroptosis may alter the signals that osteocytes transmit to remodelling surfaces, particularly RANKL/OPG-related osteoclastogenic signals (17).

Direct osteocyte evidence is available in oestrogen-deficiency, glucolipotoxic, natural-ageing, and glucocorticoid-exposure models, although the depth of validation differs among studies (17, 21, 89, 90). These models assessed combinations of iron accumulation, membrane lipid peroxidation, impaired GPX4- or SLC7A11-related defence, cell death, specific rescue, genetic intervention, and skeletal outcomes. Where System Xc^−^–GSH–GPX4 defence becomes insufficient and iron-dependent membrane phospholipid peroxidation accompanies death and ferroptosis-specific rescue, osteocyte injury can be classified more confidently as ferroptotic. The resulting RANKL-related “signal-conversion” effect has been demonstrated in the ovariectomy model and should not be generalised to every oxidative-stress setting (17).

In postmenopausal osteoporosis, ferroptotic osteocytes may enhance RANKL-mediated osteoclast formation (17). In the model reported by Jiang et al., reduced Nrf2 signalling was associated with reduced Dnmt3a expression, lower methylation of the RANKL promoter, and increased RANKL expression; restoration of this axis suppressed the osteoclastogenic signal (17). This directional Nrf2–Dnmt3a–RANKL mechanism links osteocyte ferroptosis to epigenetic regulation and osteoclastogenesis rather than leaving the conclusion at the level of nonspecific oxidative injury.

In diabetic osteoporosis, direct evidence is available in osteocytes: Yang et al. demonstrated iron accumulation, lipid peroxidation, and osteocyte death under glucolipotoxic conditions, and linked this response to HO-1 activation regulated by Nrf2 and c-JUN (21). Targeting ferroptosis or HO-1 rescued osteocyte death and ameliorated trabecular deterioration in diabetic models (21). This supports osteocyte ferroptosis in a defined diabetic context; it does not imply that every bone-cell population undergoes the same process.

More recent studies extend direct osteocyte evidence beyond diabetic and postmenopausal settings. In naturally aged mice, ATF3 upregulation increased TFR1-dependent iron uptake and suppressed SLC7A11, promoting osteocyte lipid peroxidation and cortical bone loss; pharmacological ATF3 inhibition reduced these changes (89). In glucocorticoid-induced osteoporosis, osteocyte-specific GPX4 deletion, ferroptosis-inhibitor or iron-chelator rescue, and bone-targeted PSMD14 restoration support a PSMD14–SLC7A11 mechanism (90). These data strengthen ageing- and glucocorticoid-specific conclusions but do not justify extrapolation to mechanical unloading or all osteoporosis phenotypes.

Loss of osteocyte viability can impair mechanosensation and microdamage responses, as supported by osteocyte-ablation studies (1–3, 88). However, those experiments do not identify ferroptosis as the mode of death. Accordingly, altered mechanosensation is a plausible consequence of ferroptotic damage to the osteocyte network, whereas a direct causal claim that absence of mechanical stimulation induces osteocyte ferroptosis is not supported by the evidence cited here.

Therefore, the significance of protecting osteocytes from ferroptotic injury lies not only in maintaining their survival but also in preserving remodelling communication in the models in which that coupling has been demonstrated (17, 21). Future studies should further distinguish the relative contributions of osteocyte ferroptosis, apoptosis, and nonspecific oxidative injury in each osteoporosis subtype.

### 4.3 Osteoclast ferroptosis and bone resorption

Osteoclasts are multinucleated bone-resorbing cells derived from the monocyte/macrophage lineage, and their differentiation depends primarily on M-CSF and RANKL (26, 91, 92). In osteoporosis, excessive osteoclast formation or prolonged osteoclast survival shifts bone remodelling towards resorption (91–96). This biological setting provides the rationale for testing whether ferroptosis can terminate pathological osteoclast survival and resorptive activity in defined high-resorption or iron-uptake models (34, 38).

Osteoclast differentiation and resorptive activity depend on controlled redox signalling, and moderate ROS generation supports RANKL-induced osteoclastogenesis (31, 97, 98). This physiological signalling must be distinguished from lethal iron-dependent phospholipid peroxidation. During RANKL-induced differentiation under normoxia, NCOA4-mediated ferritinophagy was reported to release ferritin-stored iron, expand the labile iron pool, and increase susceptibility to ferroptosis; hypoxia/HIF-1α suppressed autophagosome formation and ferritinophagy, thereby reducing iron release, ROS accumulation, and ferroptotic injury (34). The same study reported that pharmacological HIF-1α inhibition attenuated bone loss in ovariectomised mice (34). These data support a model-specific osteoclast mechanism and an *in vivo* pharmacological effect, but do not by themselves prove that hypoxic protection is a normal physiological adaptation or that the skeletal benefit is exclusively osteoclast mediated.

Zoledronic acid provides a treatment-related example. In a study motivated by bisphosphonate-related osteonecrosis of the jaw, zoledronic acid promoted osteoclast ferroptosis by inhibiting FBXO9-mediated p53 ubiquitination and degradation (38). The broader p53–SLC7A11 relationship provides mechanistic context, but was established outside this specific bone model (74). Accordingly, the study supports a relevant osteoclast mechanism; it does not establish that selective ferroptosis is responsible for the net clinical benefit of zoledronate in osteoporosis or that the mechanism is free of jaw-specific safety implications.

NOX4-dependent redox signalling is relevant here as a differentiation mechanism that must be distinguished from osteoclast ferroptosis. NOX4 supports osteoclastogenesis, and RANKL-induced NOX4/ROS/PERK/eIF-2α/ATF4 signalling can enhance autophagic flux and resorption-associated markers (99, 100). These findings explain how ROS may sustain osteoclast formation rather than establish ferroptotic death; they should not be used as evidence that NOX4 mediates ferroptosis in osteoclasts.

Two additional studies refine the osteoclast evidence. MZF1 deficiency enhanced RANKL-induced osteoclastogenesis while decreasing Fe²^+^, MDA, and ROS and increasing Nrf2/GPX4-related defence; because ferroptosis-specific rescue was not reported, this supports an association rather than a complete causal chain (101). In an iron-uptake/overload context, artesunate increased lipid-peroxidation products and ferroptotic mitochondrial features in differentiating osteoclasts and reduced bone loss, supporting pharmacologically induced osteoclast ferroptosis in that model (102). Neither study establishes that iron overload alone invariably causes ferroptosis in mature osteoclasts.

Osteoclast ferroptosis therefore has a context-dependent remodelling consequence. When induced in differentiating or pathologically overactive osteoclasts, it may shorten osteoclast survival and reduce excessive resorption; conversely, broad ferroptosis inhibition could preserve osteoclast survival in a high-resorption state (34, 38). This interpretation remains preclinical and should be restricted to the tested differentiation, iron-uptake, and drug-exposure models.

The therapeutic implication is selectivity: a candidate intervention would need to increase ferroptotic vulnerability specifically in pathological osteoclasts while preserving physiological remodelling and coupling. Current studies have not established a clinically translatable osteoclast-specific delivery system, an optimal induction window, or long-term net skeletal benefit, so osteoclast ferroptosis should be presented as a proposed antiresorptive mechanism rather than an established treatment strategy (34, 38).

### 4.4 Bone marrow mesenchymal stem cell ferroptosis and failure of osteogenic differentiation

BMSCs are an upstream source of the osteoblast lineage and contribute to the continuing supply of osteogenic cells. Compared with ferroptosis in mature osteoblasts, BMSC ferroptosis can reduce cell survival and weaken the osteogenic progenitor compartment, resulting in insufficient matrix production and mineralisation (4–7). Directly investigated contexts include glucocorticoid exposure, tobacco-toxin exposure, glucolipotoxic stress, erastin-induced injury, and more recent ageing-related models (20, 25, 103–106). Iron overload also impairs BMSC osteogenic commitment, but the cited iron-overload study did not by itself establish ferroptosis (12). Across directly tested models, impaired System Xc^−^–GSH–GPX4 defence, altered metabolism or proteostasis, iron dysregulation, and lipid peroxidation were associated with reduced osteogenic markers and mineralisation (20, 25, 103, 104).

Osteoporotic and diabetic bone-marrow environments can show contraction of the osteogenic compartment together with increased marrow adiposity (107). Earlier evidence did not establish that BMSC ferroptosis directly drives a lineage-allocation shift. More recent lineage-focused models now support a closer link: Steap4-driven ferroptosis was coupled to adipogenic bias in senescent BMSCs, whereas Parkin restoration limited ACSL4-dependent ferroptosis and restored osteogenic differentiation in glucocorticoid-exposed BMSCs (105, 106). These conclusions remain restricted to the reported mouse and cell models.

Ferritin-related iron homeostasis is an important upstream component of BMSC ferroptosis. Abnormalities in FTH1/FTL or enhanced NCOA4-mediated ferritinophagy can expand the labile iron pool and promote lipid peroxidation (20, 41, 42, 54). In senescent BMSCs, the ferrireductase Steap4 increased Fe²^+^ accumulation and reinforced a ROS/C/EBPβ feedback loop (105). In smoking-related osteoporosis, tobacco toxin-induced BMSC ferroptosis supports injury to the osteogenic progenitor compartment (20). A separate study of curcumin-loaded engineered exosomes reported protection of BMSCs and mitigation of smoking-related bone loss (108).

Exosome-based interventions further suggest that BMSCs at risk of ferroptosis can retain osteogenic function if injury is interrupted before terminal membrane failure. Human umbilical-cord mesenchymal stromal cell-derived exosomes preserved BMSC proliferation and osteogenesis in a glucocorticoid model through PI3K/AKT-associated suppression of lipid peroxidation (109), while curcumin-loaded engineered exosomes targeted BMSCs in a smoking-related model (108). These studies support BMSC protection, but their donor cells, cargoes, delivery approaches, and levels of ferroptosis validation should not be treated as interchangeable.

Recent lineage-focused studies strengthen BMSC evidence in ageing- and glucocorticoid-related osteoporosis. In senescent BMSCs, Steap4-driven Fe²^+^ accumulation and a ROS/C/EBPβ feedback loop promoted ferroptosis and adipogenic bias; Prrx1-lineage Steap4 deletion or DFO/ferrostatin-1 rescue restored osteogenic outcomes and attenuated senile bone loss (105). In glucocorticoid-exposed BMSCs, Parkin-mediated K48-linked ubiquitination and degradation of ACSL4 limited ferroptosis; bone-targeted Parkin mRNA delivery restored osteogenesis and reduced bone loss in mice (106). These findings add cell- and subtype-specific causal evidence without establishing clinical efficacy.

BMSC ferroptosis reduces the cellular source available to enter the osteogenic lineage. It may also favour adipogenic allocation where that shift has been tested directly, as in the senescent Steap4 model (105). Therapeutic strategies need to protect mature osteoblasts, osteocytes, and the BMSC compartment simultaneously, with particular emphasis on maintaining BMSC viability, osteogenic commitment, and iron–lipid homeostasis.

### 4.5 Integrated cell-type differences and evidence ranking

Evidence remains uneven across bone-remodelling cell populations. Osteoblast studies provide a broad combination of direct mechanisms, ferroptosis-specific rescue, and skeletal phenotypes (8, 18, 19, 66–68, 75, 79). Osteocyte studies now include direct mechanisms in oestrogen-deficiency, diabetes, ageing, and glucocorticoid models, with genetic intervention and *in vivo* outcomes in selected studies (17, 21, 89, 90). New lineage-focused ageing and glucocorticoid studies strengthen BMSC evidence through genetic or targeted rescue (105, 106), whereas osteoclast evidence remains more model dependent despite additional genetic-association and pharmacological-induction studies (34, 38, 101, 102).

The pathological consequences also differ: osteoblast ferroptosis impairs matrix formation and mineralisation; BMSC ferroptosis reduces survival and osteogenic progenitor capacity and can favour adipogenic allocation in defined models; osteocyte ferroptosis alters remodelling communication and contributes to cortical or trabecular deterioration in specific contexts; and osteoclast ferroptosis may shorten the survival of pathological resorptive cells. The net skeletal effect is therefore determined by cell identity, disease context, and turnover state rather than by ferroptosis alone (17, 18, 20, 21, 25, 34, 38, 88).

This distinction supports a cell-selective therapeutic strategy that protects osteoblasts, osteocytes, and BMSCs while increasing ferroptotic susceptibility only in pathologically overactive osteoclasts. This framework also requires preservation of coupling: BMSCs replenish osteoblasts, osteoblast-lineage cells support matrix formation and OPG-related regulation, and osteocyte-derived RANKL controls osteoclast recruitment and activity (1–7, 110). Consequently, lowering resorption alone is insufficient to establish a favourable net effect; formation, resorption, and their coordination should be assessed together. These cell-specific evidence levels and principal limitations are summarised in **Table 2**.

**Table 2:** Highest evidence level, principal remodelling outcome, and key limitations of ferroptosis in four bone-remodelling cell populations.

Cell type	Highest level	Representative evidence	Principal bone-remodelling outcome	Key limitations
Osteoblasts	Level A	NOX4-driven ferroptosis under iron overload and a genetic-pharmacological causal chain involving DNMT/GPX4; diabetic and ageing models provide complementary support (8, 18, 19, 66–68, 75, 81).	Impaired osteogenic differentiation, matrix formation, and mineralisation.	Insufficient human causal evidence, cross-model replication, and bone-targeted intervention data.
Osteocytes	Level A	Genetic GPX4 models, ovariectomy phenotypes, diabetic glucolipotoxic rescue, natural-ageing ATF3/TFR1 intervention, and glucocorticoid PSMD14/SLC7A11 studies (17, 21, 89, 90).	Reduced osteocyte survival and altered RANKL-related remodelling communication.	Evidence is still preclinical; human causal validation and independent replication across subtypes remain limited.
Osteoclasts	Level B	RANKL/HIF-1α/NCOA4 regulation, MZF1-associated Nrf2/GPX4 changes, artesunate induction in iron-overload bone loss, and zoledronate-induced ferroptosis in a jaw-osteonecrosis context (34, 38, 101, 102).	Ferroptosis in pathologically overactive osteoclasts may limit bone resorption.	Ferroptosis-specific rescue is incomplete in the MZF1 study; clinically translatable osteoclast-specific delivery and long-term coupling data are lacking.
BMSCs	Level A	Glucocorticoid, tobacco-toxin, glucolipotoxic, ageing, and erastin models include rescue; Steap4 lineage deletion and bone-targeted Parkin restoration add model-specific *in vivo* causal evidence (20, 25, 103–106, 108, 109).	Reduced survival and osteogenic capacity; the senescent Steap4 model also supports adipogenic bias, whereas the glucocorticoid Parkin model supports restored osteogenesis.	High-level evidence is concentrated in recent mouse models; human causality, replication, delivery safety, and durable lineage outcomes remain unestablished.

## 5 Ferroptosis-related signalling pathways in osteoporosis

This section organises pathways according to their positions within the ferroptotic cascade. Nrf2/HO-1/SLC7A11/GPX4 represents defence against lipid peroxidation; NOX4 represents amplification of reactive oxygen species; Wnt/β-catenin represents the osteogenic programme; DNMT/GPX4 and METTL3/ASK1-p38 represent epigenetic or epitranscriptomic regulation; HIF-1α/NCOA4 and FtMt/PINK1/Parkin connect ferritinophagy and mitochondrial iron homeostasis; and PSMD14/SLC7A11 and Parkin/ACSL4 illustrate proteostasis-dependent defence in osteocytes and BMSCs, respectively. PI3K/AKT/mTOR and AMPK/Nrf2 are therapeutically relevant metabolic regulators (**Figure 5**). Pathway inclusion is based on the specific ferroptosis experiments cited below. General ROS, inflammatory, autophagic, or metabolic functions are discussed only to distinguish them from validated ferroptosis mechanisms and are not evidence of ferroptosis by themselves.

**Figure 5:**
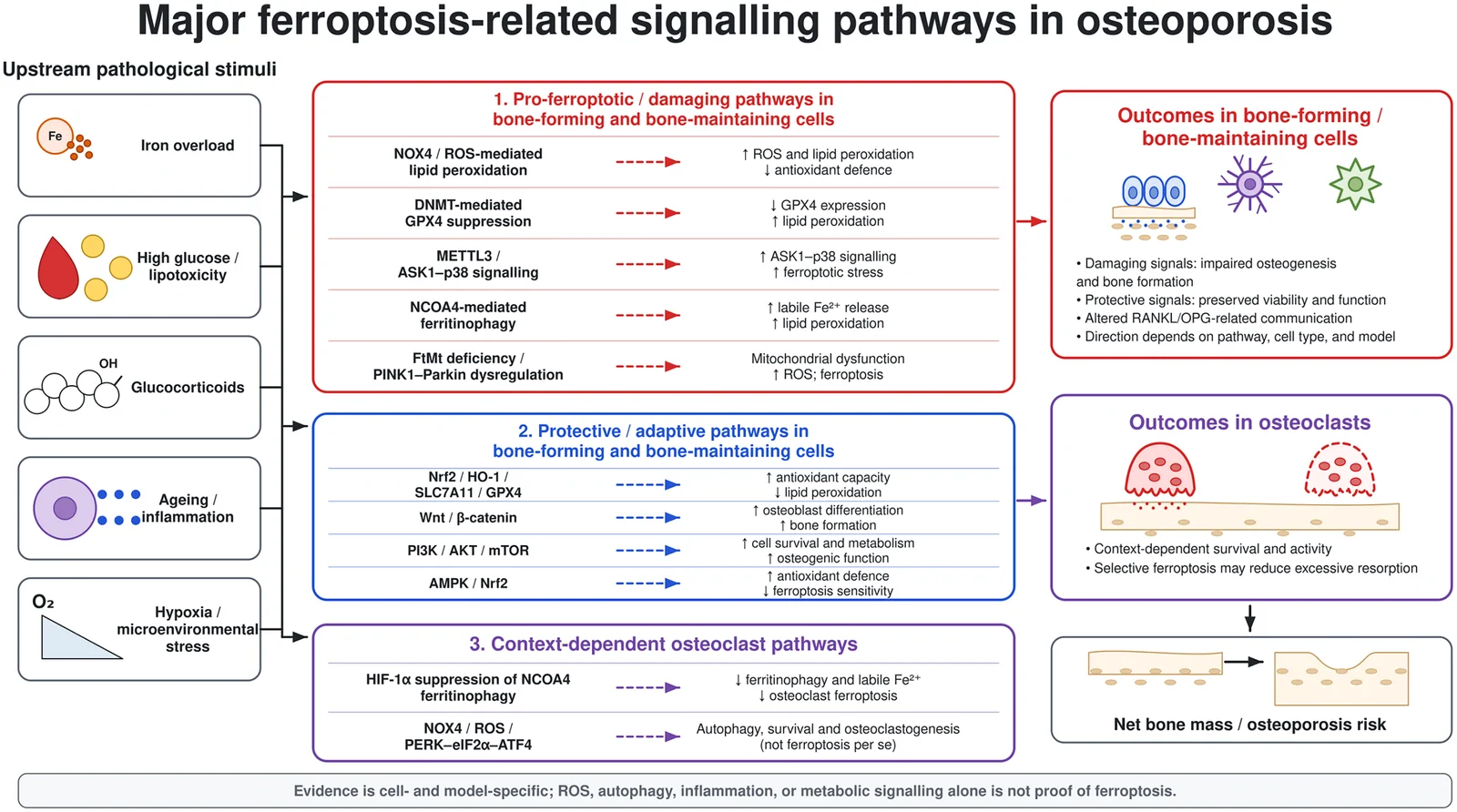
Major ferroptosis-related signalling pathways involved in osteoporosis. Cell colours follow **Figure 4**. Red damaging and blue protective pathways converge on bone-forming or bone-maintaining cell functions but have opposite directional effects; the purple pathway connects specifically to osteoclast outcomes. The NOX4/PERK/autophagy branch denotes osteoclast differentiation rather than demonstrated ferroptosis. The net skeletal outcome depends on pathway direction, cell type, and disease context.

### 5.1 Nrf2/HO-1/SLC7A11/GPX4 pathway

The Nrf2/HO-1/SLC7A11/GPX4 axis is a general anti-ferroptotic defence network (24, 64, 73, 111–113). Its involvement in bone cells has been demonstrated in defined osteoblast, osteocyte, and BMSC models (17, 19, 25, 68, 81). Under oxidative or electrophilic stress, Nrf2 can induce SLC7A11, GSH metabolism, GPX4-related defences, and iron-handling genes, thereby increasing the capacity to clear phospholipid hydroperoxides. The direction and causal depth of this response must nevertheless be established separately in each cell and disease model.

The effect of Nrf2/HO-1 signalling is model dependent rather than uniformly protective. In high-glucose-exposed osteoblasts, Nrf2/HO-1 activation was associated with protection from ferroptotic injury (67). In glucolipotoxic osteocytes, however, HO-1 activity promoted iron accumulation and ferroptosis, and HO-1 inhibition was protective (21). Because haem degradation can release iron, general biochemical studies provide a plausible explanation for this divergent effect, but do not establish a universal dose threshold in bone cells (114). The pathway should therefore be described according to the specific cell type and experimental context.

In diabetic osteoporosis, interventions such as melatonin and Maresin 1 can attenuate high-glucose-induced osteoblast ferroptosis through Nrf2/HO-1 or Nrf2/SLC7A11/GPX4 signalling (19, 67). In osteocytes, Nrf2 may influence osteoclastogenesis through Dnmt3a/RANKL-related mechanisms (17), whereas glucocorticoid exposure can destabilise SLC7A11 by disrupting PSMD14-mediated deubiquitination (90). In differentiating osteoclasts, MZF1 loss was associated with increased Nrf2/GPX4 and reduced iron/lipid-oxidation markers, but the absence of ferroptosis-specific rescue limits the causal strength of that study (101).

### 5.2 NOX4/ROS/lipid peroxidation pathway

NOX4 is an important node linking iron overload, ROS amplification, and lipid peroxidation (18, 99). Its ROS-generating capacity largely depends on its expression level, making it particularly relevant in sustained oxidative stress (18, 99).

In iron-overload models, Zhang et al. connected iron-dependent derepression of NOX4 to lipid-peroxide accumulation and mitochondrial injury in osteoblasts (18). Ferrostatin-1 and deferoxamine prevented the bone loss observed in iron-accumulating Hepc1-deficient mice, providing pharmacological rescue beyond changes in ROS alone (18). These findings support NOX4-triggered osteoblast ferroptosis in this setting, while the human skeletal observations remain associative.

NOX4 can also promote osteoclast differentiation through RANKL-induced ROS signalling; increased PERK/eIF-2α/ATF4 signalling and autophagic flux are differentiation-supportive mechanisms, resulting in increases in markers such as TRAP, Cath K, and MMP-9 and in resorption lacunae (99, 100). Because moderate NOX4–ROS signalling also participates in early osteogenesis (115), a candidate intervention would need to limit pathological overactivation without abolishing physiological redox signalling. The osteoclast studies cited here establish differentiation-related ROS signalling, not NOX4-dependent osteoclast ferroptosis; the osteoblast conclusion cannot be transferred across these cell types.

### 5.3 Wnt/β-catenin pathway

Canonical Wnt/β-catenin signalling has established roles in osteoblast differentiation, mineralisation, and the regulation of osteogenic genes such as RUNX2, ALP, and OCN (48, 85–87, 110, 116–119). Its relevance here is model specific: Wnt signalling can determine whether osteoblasts maintain their osteogenic programme during iron-overload and lipid-peroxidation stress, but its general developmental functions do not independently demonstrate ferroptosis.

In iron-overloaded osteoblasts, pharmacological Wnt activation reduced ROS and lipid peroxidation and prevented ferroptosis without reducing iron overload, thereby restoring osteogenic differentiation (87). This finding places the protective Wnt effect downstream of iron accumulation in the tested model; it does not imply that Wnt activation is merely a post-death transcriptional repair mechanism or that global Wnt activation would be appropriate across cell types and developmental stages.

### 5.4 DNMT/GPX4 epigenetic pathway

The DNMT/GPX4 pathway indicates that osteoblast ferroptosis can be driven by stable transcriptional repression (75). When DNMT1, DNMT3a, and DNMT3b are abnormally elevated, hypermethylation of the GPX4 promoter can suppress GPX4 expression, reduce phospholipid-hydroperoxide clearance capacity, and lower the ferroptosis threshold in osteoblasts (75).

This mechanism lies upstream of GPX4 activity: if GPX4 is directly inactivated, DNMT inhibition is unlikely to fully restore defence; if GPX4 downregulation results from promoter hypermethylation, DNMT inhibition may reactivate GPX4 and reduce osteoblast ferroptosis (16, 75). Future studies should identify the upstream causes of DNMT abnormalities and achieve more selective restoration of GPX4 in bone-forming cells.

### 5.5 HIF-1α/NCOA4/ferritinophagy pathway

The HIF-1α/NCOA4/ferritinophagy pathway is primarily used to explain osteoclast ferroptosis (34). NCOA4 mediates ferritinophagy, releases ferritin-stored iron, and expands the labile iron pool; during RANKL-induced differentiation, this process can increase ferroptotic pressure in osteoclasts (34, 41, 42, 54, 55).

Hypoxia/HIF-1α suppressed RANKL-induced ferritinophagy, reduced iron release and ROS accumulation, and protected differentiating osteoclasts from ferroptosis (34). Pharmacological HIF-1α inhibition also attenuated bone loss in ovariectomised mice (34). These findings support a candidate strategy for increasing osteoclast ferroptotic susceptibility in a high-resorption model; whether the hypoxic response represents physiological adaptation during normal remodelling and whether the *in vivo* effect is osteoclast specific remain unresolved.

The preclinical implication is therefore not broad HIF-1α inhibition, but identification of osteoclast-specific downstream targets that could increase ferroptotic susceptibility in overactive osteoclasts while avoiding effects on angiogenesis, osteoblast adaptation, and normal bone remodelling (34).

### 5.6 FtMt/PINK1/Parkin-mediated mitophagy pathway

The FtMt/PINK1/Parkin axis primarily explains dysregulated mitochondrial iron metabolism in osteoblasts during diabetic osteoporosis (8). FtMt is localised within mitochondria and can buffer excess mitochondrial iron; when FtMt is insufficient, mitochondrial iron more readily participates in ROS generation, increasing lipid peroxidation and ferroptotic pressure in osteoblasts (8).

Under high-glucose stress, FtMt deficiency can activate ROS/PINK1/Parkin-mediated mitophagy. Mitophagy is itself a quality-control mechanism, but under sustained high glucose and excessive mitochondrial iron loading, it may reflect failed compensation and coexist with aggravated ferroptosis (8). The focus of intervention should be restoration of mitochondrial iron homeostasis and appropriate quality control, rather than simply inhibiting or activating mitophagy.

Parkin also has a distinct, mitophagy-independent role in glucocorticoid-exposed BMSCs: it directly targets ACSL4 for K48-linked ubiquitination and proteasomal degradation, thereby reducing PUFA-phospholipid peroxidation and ferroptosis (106). This BMSC mechanism should not be conflated with the FtMt/ROS/PINK1/Parkin mitophagy pathway described in diabetic osteoblasts (106).

### 5.7 METTL3/ASK1-p38 pathway

The METTL3/ASK1-p38 pathway provides an epitranscriptomic mechanism for diabetic osteoporosis (66). In high-glucose/high-lipid osteoblast models, METTL3-mediated m6A regulation was linked to ASK1-p38 activation, iron accumulation, lipid peroxidation, reduced SLC7A11/GPX4 defence, and impaired osteogenic differentiation (66). General ASK1 and m6A studies provide biochemical context for stress-kinase and epitranscriptomic regulation, but do not independently validate this osteoblast-ferroptosis mechanism (120–122).

The value of this axis lies in its disease specificity: it explains how the diabetic bone microenvironment can cause sustained osteoblast injury through RNA modification and stress kinases rather than solely through general oxidative stress (66). Because METTL3 has a broad range of targets, future interventions should focus on ASK1-p38-related m6A regulatory components rather than nonspecific inhibition of METTL3.

### 5.8 PI3K/AKT/mTOR and AMPK/Nrf2 pathways

PI3K/AKT/mTOR and AMPK/Nrf2 are more appropriately regarded as therapeutic regulatory frameworks (25). The former is oriented towards cell survival, protein synthesis, and maintenance of osteogenesis and is commonly used to explain protection of BMSCs and osteoblasts under glucocorticoid stress; melatonin alleviates BMSC ferroptosis through this pathway, suggesting that restoration of survival-metabolic signalling can improve the anti-ferroptotic capacity of bone-forming cells (25).

AMPK/Nrf2 is associated with adaptation to energy stress and resistance to lipid peroxidation (73, 111, 112, 123). AMPK can inhibit ACC activity and reduce readily peroxidisable lipid substrates, whereas Nrf2 can induce SLC7A11, glutathione metabolism, GPX4-dependent defences, and iron-handling genes. Metformin and β-caryophyllene have been reported to attenuate ferroptosis and bone loss under defined metabolic-stress conditions through AMPK/Nrf2-related mechanisms (81, 104). However, AMPK is not uniformly anti-ferroptotic: tobacco-toxin exposure has also been linked to AMPK activation and NCOA4-dependent ferritinophagy in BMSCs (20). The direction of effect therefore requires model-specific mechanistic and rescue evidence.

### 5.9 Evidence stratification and priority validation directions

The maturity of these pathways differs substantially. DNMT/GPX4 and NOX4 in osteoblasts, GPX4- and PSMD14/SLC7A11-related mechanisms in osteocytes, and the Steap4 and Parkin/ACSL4 mechanisms in BMSCs have relatively complete causal chains within their tested models (17, 18, 75, 90, 105, 106). Most other metabolic regulatory and mitochondrial pathways have level B evidence, while the osteoclast MZF1/Nrf2/GPX4 relationship remains association-dominant because ferroptosis-specific rescue was not reported (101). Direct evidence for FSP1-CoQ10, DHODH-CoQ10, and GCH1-BH4 in bone tissue remains primarily level C. Detailed grading is summarised in **Table 3**.

**Table 3:** Evidence grading of major ferroptosis-related pathways in osteoporosis.

Pathway/mechanism	Principal cell or model	Level	Evidence basis	Major limitation
NOX4/ROS/lipid peroxidation	Iron-overloaded osteoblasts	Level A	Genetic or pharmacological inhibition, ferroptosis rescue, and animal skeletal phenotypes form a relatively complete causal chain (18).	Human causal and precise bone-targeting evidence is lacking.
DNMT/GPX4	Osteoblasts/ovariectomy	Level A	DNMT knockdown, pharmacological reversal, osteoblast-specific Gpx4 genetic models, and *in vivo* skeletal phenotypes (75).	Upstream triggers and long-term epigenetic safety remain unclear.
Nrf2/Dnmt3a/RANKL, PSMD14/SLC7A11, and GPX4	Osteocytes/postmenopausal and glucocorticoid models	Level A	GPX4 genetics and co-culture validate the postmenopausal pathway; GPX4 conditional deletion, specific rescue, and PSMD14 restoration support the glucocorticoid pathway (17, 90).	Limited external replication and human evidence.
Wnt/β-catenin	Iron-overloaded osteoblasts	Level B	In iron-overloaded osteoblasts, Wnt activation reduced ROS and lipid peroxidation, prevented ferroptosis without lowering iron overload, and restored osteogenic differentiation (87).	The evidence is model specific; Wnt is not a ferroptosis-specific pathway and global activation is stage and cell-type dependent.
FtMt/PINK1/Parkin mitophagy and Parkin/ACSL4 ubiquitination	Diabetic osteoblasts; glucocorticoid-exposed BMSCs	Level A (highest model-specific evidence)	FtMt links mitochondrial iron and mitophagy in diabetic osteoblasts; Parkin-mediated ACSL4 degradation has genetic, rescue, targeted-delivery, and skeletal evidence in glucocorticoid-exposed BMSCs (8, 106).	The two Parkin-related mechanisms occur in different cells and disease contexts; neither has clinical validation.
HIF-1α/NCOA4/ferritinophagy	Osteoclasts	Level B	RANKL/hypoxia cellular mechanisms and pharmacological HIF-1α inhibition in ovariectomised mice support the pathway (34).	Osteoclast-specific *in vivo* causality, physiological versus pathological directionality, and clinical efficacy remain unestablished.
METTL3/ASK1-p38	Diabetic osteoblasts	Level B	Mechanistic and animal evidence links m6A regulation, stress kinases, ferroptosis, and bone loss (66).	METTL3 has broad targets; specificity and reproducibility require validation.
Nrf2-related pathways, PI3K/AKT/mTOR, AMPK/Nrf2, and Steap4/Fe²^+^–ROS/C/EBPβ	Osteoblasts/BMSCs	Level A–B, depending on model	Multiple interventions reduce ferroptosis-related phenotypes; Steap4 lineage deletion and DFO/ferrostatin-1 rescue provide stronger ageing-BMSC causality (19, 25, 67, 68, 81, 104, 105).	General antioxidant, pro-survival, or metabolic effects must be excluded.
Candidate defence mechanisms including FSP1, DHODH, and GCH1	Candidate skeletal mechanisms	Level C	Core mechanisms have been established mainly in non-skeletal systems; the selenoprotein nature of GPX4 is a supplementary variable, but direct skeletal causal evidence remains limited (25, 44–47, 76–78).	These mechanisms cannot yet be considered established therapeutic targets for osteoporosis.

Direct skeletal evidence for FSP1-CoQ10, DHODH-CoQ10, and GCH1-BH4 remains limited. These pathways should be prioritised as candidates for validation rather than presented as therapeutic targets of equal maturity to pathways with established causal chains in bone cells. The selenoprotein nature of GPX4 can be considered as a supplementary mechanistic variable, but it does not constitute an independent therapeutic theme. Priority experiments should include cell-type-specific intervention, ferroptosis-specific rescue, membrane lipidomics, and *in vivo* skeletal phenotyping rather than relying solely on changes in pathway-protein expression.

## 6 Ferroptosis across osteoporosis subtypes

Osteoporosis is not a disease of a single pathway. Although reduced bone mass and deterioration of bone microarchitecture are common outcomes, upstream causes differ among postmenopausal, diabetic, glucocorticoid-induced, ageing-related, and iron-overload-related osteoporosis, as well as inflammatory bone loss (8, 13, 14, 17, 19–21, 25, 27–32, 66–68, 79, 124–129). Direct ferroptosis evidence is available in several defined subtypes, but the affected cells, initiating stresses, and strength of causal validation differ. For inflammatory bone loss, current evidence more securely establishes cytokine, ROS, and RANKL biology than ferroptosis itself. Ferroptosis should therefore be interpreted according to disease context rather than assumed to be a uniform mechanism across all subtypes.

The dominant upstream context differs among disease types. Oestrogen deficiency has been linked to osteocyte ferroptosis and osteoblast GPX4 dysregulation in ovariectomy models (17, 75). Diabetic models involve glucolipotoxicity, mitochondrial iron dysregulation, Nrf2-related signalling, and epitranscriptomic stress in osteoblast-lineage cells and osteocytes (8, 19, 21, 66, 67, 81, 104). Glucocorticoid models now directly implicate BMSCs, osteoblasts, and osteocytes through PI3K/AKT/mTOR, Parkin/ACSL4, extracellular-vesicle, and PSMD14/SLC7A11 mechanisms (25, 79, 82, 90, 106, 109). Ageing-related evidence includes D-galactose/VDR osteoblast experiments, natural-ageing osteocyte ATF3/TFR1 evidence, and Steap4-dependent ferroptosis in senescent BMSCs (68, 89, 105). Iron-overload models provide direct osteoblast evidence and a pharmacological osteoclast-induction model (18, 87, 102).

### 6.1 Postmenopausal osteoporosis

Postmenopausal osteoporosis is driven primarily by oestrogen deficiency, oxidative stress, and enhanced bone resorption (29, 30, 32). Oestrogen withdrawal disrupts iron metabolism and increases oxidative injury in bone (30, 32, 130). In this context, ferroptosis is particularly relevant to osteocytes because osteocytes regulate bone remodelling through their dual effects on osteoblasts and osteoclasts. Therefore, ferroptotic injury in osteocytes may affect skeletal homeostasis beyond the loss of osteocyte viability itself (1–3, 17).

A key mechanism in postmenopausal osteoporosis is the conversion of osteocyte ferroptosis into osteoclastogenic signalling. In the cited model, reduced Nrf2 signalling was associated with reduced Dnmt3a, reduced methylation of the RANKL promoter, and increased RANKL expression; restoration of the pathway suppressed RANKL-related osteoclastogenesis (17). This directional Nrf2–Dnmt3a–RANKL mechanism links oestrogen deficiency, osteocyte ferroptosis, epigenetic regulation, and osteoclast-mediated bone resorption.

Osteoblast-lineage cells may also be affected in postmenopausal osteoporosis. Oestrogen deficiency is associated with increased oxidative stress, reduced antioxidant defence, and impaired bone formation (29, 30, 32). DNMT-mediated suppression of GPX4 may contribute to osteoblast ferroptosis by reducing the capacity to detoxify phospholipid hydroperoxides. In this context, GPX4 suppression does not merely represent a downstream marker of oxidative injury; it may actively lower the threshold for ferroptotic osteoblast injury and reduce bone formation (75).

Osteoclasts should be interpreted differently. Bone resorption is generally excessive in postmenopausal osteoporosis. If HIF-1α-mediated protection from ferroptosis prolongs osteoclast survival, it may indirectly support bone resorption. Conversely, increasing the ferroptotic vulnerability of overactive osteoclasts may help suppress bone loss (34, 38). Therefore, ferroptosis-targeted strategies in postmenopausal osteoporosis should aim to protect osteocytes and osteoblast-lineage cells while avoiding excessive preservation of osteoclast survival.

### 6.2 Diabetic osteoporosis

Diabetic osteoporosis is characterised by hyperglycaemia, disordered lipid metabolism, advanced glycation end products, mitochondrial dysfunction, and impaired bone quality (8, 21, 107, 131, 132). Compared with postmenopausal osteoporosis, diabetic osteoporosis has a stronger metabolic component. In this context, direct ferroptosis evidence has been obtained in defined glucolipotoxic bone-cell models that combine iron dysregulation, membrane lipid peroxidation, impaired defence, death, and intervention responses; metabolic stress or ROS accumulation alone remains insufficient for classification (8, 19, 21, 66, 67, 81).

Osteoblasts are major targets of ferroptotic injury in diabetic osteoporosis. High-glucose or high-glucose/high-lipid conditions can produce iron dysregulation, membrane lipid peroxidation, loss of GPX4-related defence, and ferroptotic death, leading to reduced osteoblast viability, impaired differentiation, and defective mineralisation in the tested models (19, 66, 67, 81). The Nrf2/HO-1 and Nrf2/SLC7A11/GPX4 pathways may protect osteoblasts by restoring antioxidant capacity and limiting lipid-peroxide accumulation. Melatonin and Maresin 1 are representative interventions reported to inhibit high-glucose-induced osteoblast ferroptosis through Nrf2-related signalling (19, 67).

Mitochondrial mechanisms are also prominent in diabetic osteoporosis. FtMt deficiency can aggravate dysregulated mitochondrial iron metabolism and promote osteoblast ferroptosis through ROS/PINK1/Parkin-mediated mitophagy. This mechanism indicates that mitochondria are not only injured during ferroptosis but may also act as upstream amplifiers of ferroptotic stress when mitochondrial iron buffering fails (8). Because osteoblast differentiation depends heavily on mitochondrial function, mitochondrial iron handling is particularly important in diabetic bone loss (8).

Epitranscriptomic regulation further expands the mechanisms of diabetic osteoporosis. High-glucose/high-lipid stress may activate METTL3-mediated m6A modification and the ASK1-p38 pathway, thereby increasing ferroptosis susceptibility in osteoblasts. This indicates that diabetic osteoporosis is not merely a disease of oxidative stress; it may also involve RNA-level regulation of stress-kinase signalling (66). In osteoblasts and BMSCs, AMPK/Nrf2 activation may provide a protective metabolic-redox response by reducing oxidative stress, supporting antioxidant defence, and lowering lipid-peroxidation pressure (81, 104).

Osteocytes are another important target in diabetic osteoporosis. Glucolipotoxicity can induce osteocyte ferroptosis and disrupt osteocyte-mediated regulation of bone remodelling (21). Because osteocytes coordinate communication between osteoblasts and osteoclasts, ferroptotic osteocyte injury may contribute to both impaired bone formation and increased bone resorption. Therefore, diabetic osteoporosis may involve ferroptosis in multiple cell types, but the predominant pattern is metabolic stress-induced injury to osteoblasts, osteocytes, and BMSCs (8, 19, 21, 66, 67, 81, 104).

### 6.3 Glucocorticoid-induced osteoporosis

Glucocorticoid-induced osteoporosis is caused primarily by prolonged or excessive glucocorticoid exposure (27, 28). Its pathological features include impaired osteogenic differentiation of BMSCs, reduced osteoblast formation, osteocyte dysfunction or death, and disturbed bone remodelling (27, 28). Ferroptosis provides one mechanism by which glucocorticoids can convert oxidative, metabolic, and proteostatic stress into injury of BMSCs, osteoblasts, and osteocytes (25, 79).

BMSCs, osteoblast-lineage cells, and osteocytes are directly investigated cellular targets in glucocorticoid-induced osteoporosis (25, 27, 28, 79, 82, 90, 106, 109). Evidence now includes ferroptosis-specific rescue and genetic or targeted intervention in BMSCs and osteocytes, while extracellular-vesicle osteoblast studies provide complementary evidence of differing mechanistic depth. When iron-dependent membrane lipid peroxidation and insufficient SLC7A11–GSH–GPX4 defence accompany death and appropriate rescue, the injury can be described as ferroptotic; glucocorticoid-induced ROS elevation alone remains insufficient.

Ferritinophagy-dependent ferroptosis may participate in glucocorticoid-induced osteoblast injury. Vascular endothelial cell-derived exosomes were reported to restrict ferritinophagy, iron release, and ferroptotic injury in glucocorticoid-exposed osteoblasts (79). By contrast, endothelial progenitor cell-derived extracellular vesicles improved osteogenic and skeletal outcomes together with redox and SLC7A11/GPX4-associated readouts, but the study did not fully establish ferritinophagy or ferroptosis as the causal death mechanism (82). The two studies therefore support different levels of mechanistic certainty and should not be merged into a single ferritinophagy claim.

Several distinct mechanisms are relevant in this subtype. Melatonin inhibited glucocorticoid-induced BMSC ferroptosis through PI3K/AKT/mTOR signalling (25), while Parkin-mediated K48-linked ubiquitination of ACSL4 limited BMSC lipid peroxidation and bone-targeted Parkin mRNA delivery restored osteogenesis in mice (106). In osteocytes, PSMD14 stabilised SLC7A11; osteocyte-specific GPX4 deletion and rescue with ferroptosis inhibitors or an iron chelator supported a causal role in glucocorticoid bone loss (90). These findings support cell-specific preclinical targets, not clinical treatment recommendations.

### 6.4 Ageing-related osteoporosis

Ageing-related osteoporosis is associated with chronic oxidative stress, impaired osteoblast survival, altered osteocyte viability, and declining BMSC osteogenic capacity (30, 124). Direct ferroptosis evidence now comes from three complementary settings: D-galactose-induced accelerated-ageing and VDR-knockout osteoblast models (68), naturally aged mice with an osteocyte ATF3/TFR1 mechanism (89), and senescent BMSCs with Steap4-dependent Fe²^+^ accumulation and genetic or pharmacological rescue (105). These models strengthen ageing-related evidence but do not constitute direct proof in natural human ageing.

Osteoblasts, osteocytes, and BMSCs are now directly supported targets in distinct ageing models. In osteoblasts, VDR/Nrf2/GPX4 signalling connects lipid peroxidation, senescence, and skeletal phenotypes (68). In BMSCs, Steap4-driven Fe²^+^ accumulation and a ROS/C/EBPβ feedback loop promoted ferroptosis and adipogenic bias; Prrx1-lineage Steap4 deletion or DFO/ferrostatin-1 rescue improved osteogenic and skeletal outcomes (105).

Osteocytes are long-lived cells exposed to cumulative oxidative and metabolic stress. In naturally aged mice, ATF3 upregulation increased TFR1-dependent iron uptake and suppressed SLC7A11, thereby promoting osteocyte lipid peroxidation and cortical bone loss; ATF3 inhibition reduced these changes (89). This evidence supports an ageing-specific ferroptosis mechanism, but it does not show that mechanical unloading itself induces osteocyte ferroptosis or establish a validated human target.

Therapeutically, ageing-related osteoporosis may require coordinated protection of osteoblasts, osteocytes, and the BMSC compartment rather than targeting a single pathway. Candidate approaches include restoration of VDR/Nrf2/GPX4 defence, interruption of the ATF3/TFR1 iron-uptake mechanism, or control of Steap4-dependent Fe²^+^ accumulation (68). All remain preclinical, and excessive antioxidant or anti-ferroptosis intervention could interfere with physiological redox signalling and normal remodelling.

### 6.5 Iron-overload-related osteoporosis

Iron-overload-related osteoporosis provides the most direct disease context linking iron metabolism to ferroptosis. Excess iron expands the labile iron pool, increases Fenton chemistry, and accelerates phospholipid peroxidation (9, 10, 12–14, 49, 50, 52, 53). In osteoblasts, iron-dependent derepression of NOX4 drove ferroptosis, and ferrostatin-1 or deferoxamine prevented bone loss in Hepc1-deficient mice (18). Wnt activation reduced lipid peroxidation and prevented osteoblast ferroptosis without lowering iron overload (87). A separate study used artesunate to induce ferroptotic features in differentiating osteoclasts and reduce iron-overload-associated bone loss; this supports a pharmacological osteoclast mechanism, not a claim that iron overload alone invariably kills mature osteoclasts (102).

Osteoblasts are the principal cells affected in iron overload-related osteoporosis. Excess iron can suppress osteoblast viability, reduce osteogenic differentiation, and impair mineralisation (9, 10, 18, 52). NOX4 appears to be a key amplification node in this process. Iron overload may increase NOX4 expression, leading to NADPH consumption, ROS generation, mitochondrial dysfunction, and lipid-peroxide accumulation. Thus, iron overload does not act only through passive Fenton chemistry; it can also activate enzymatic ROS production and thereby sustain ferroptotic stress in osteoblasts (9, 10, 18, 52).

The Wnt/β-catenin pathway is another important mechanism in iron overload-related osteoporosis. Canonical Wnt signalling supports osteoblast differentiation and mineralisation. Iron overload can inhibit Wnt/β-catenin signalling and thereby weaken the osteogenic programme (87). Activation of Wnt signalling, use of ferroptosis inhibitors, or iron chelation may restore osteoblast differentiation by reducing ROS and lipid peroxidation. This supports preservation of osteoblast differentiation in the tested iron-overload model, rather than designation of all Wnt signalling as a ferroptosis-specific pathway (87).

Preclinical strategies for iron-overload-related osteoporosis may combine correction of pathological iron loading with inhibition of ferroptotic lipid damage in bone-forming cells, while selectively reducing pathological osteoclast activity. Deferoxamine can reduce excessive iron burden, ferrostatin-1 can limit propagation of lipid peroxidation, and artesunate provides a pharmacological osteoclast-induction example (9, 18, 35, 36, 102). Iron chelation must nevertheless be titrated because physiological iron is required for skeletal homeostasis; artesunate has not been established as an osteoporosis therapy.

### 6.6 Inflammatory bone loss

Inflammatory bone loss is driven by cytokine stimulation, oxidative stress, and increased osteoclastogenesis and may occur in rheumatoid arthritis, periodontitis, infection-associated bone destruction, and related conditions (31, 32, 91–98, 125–127). These studies establish inflammatory ROS and RANKL biology, but do not by themselves demonstrate iron-dependent membrane phospholipid peroxidation or ferroptosis-specific rescue in a defined bone-cell population.

Inflammatory stress can reduce osteoblast differentiation and disrupt the balance between bone formation and resorption (128, 129). Progression from this oxidative injury to ferroptosis is biologically plausible if iron-dependent lipid peroxidation overwhelms defence systems, but the cited inflammatory bone-loss studies do not directly establish that transition. Accordingly, inflammatory oxidative injury should not be labelled as BMSC or osteoblast ferroptosis without model-specific validation.

Osteocytes can regulate inflammatory bone remodelling through RANKL/OPG-related communication (1–3, 91–95, 127). The Nrf2–Dnmt3a–RANKL link reported in ferroptotic osteocytes was demonstrated in an ovariectomy model rather than in the inflammatory diseases listed here (17). Its relevance to inflammatory bone loss is therefore a testable hypothesis, not direct evidence that inflammatory stimuli induce osteocyte ferroptosis.

A possible therapeutic implication is that anti-inflammatory or antiresorptive treatment might need to be combined with cell-specific protection from ferroptosis if a complete ferroptotic mechanism is demonstrated in a particular inflammatory model. At present, broad ferroptosis inhibition cannot be recommended from the cited evidence because its effects on immune regulation, host defence, and osteoclast survival remain insufficiently characterised.

### 6.7 Summary

Osteoporosis subtypes converge on imbalanced remodelling but differ in the location and maturity of their ferroptosis evidence. Postmenopausal models provide direct osteocyte RANKL-communication and osteoblast GPX4 evidence (17, 75); diabetic models implicate osteoblasts, osteocytes, and BMSCs (8, 19, 21, 66, 67, 81, 104); glucocorticoid models now include BMSC, osteoblast, and osteocyte mechanisms (25, 79, 82, 90, 106, 109); ageing-related evidence includes osteoblast, osteocyte, and lineage-focused BMSC models (68, 89, 105); and iron-overload models provide direct osteoblast evidence plus a pharmacological osteoclast-induction study (18, 87, 102). Inflammatory bone loss still has a less complete ferroptosis-specific causal chain. **Table 4** summarises these boundaries.

**Table 4:** Evidence levels and interpretive boundaries of ferroptosis across osteoporosis subtypes.

Osteoporosis subtype	Principal affected cells	Level	Evidence basis	Interpretive boundary
Postmenopausal osteoporosis	Osteocytes, osteoblasts, and osteoclasts	Level A	Osteocyte GPX4 genetic models, RANKL communication, and the osteoblast DNMT/GPX4 mechanism provide *in vivo* causal support (17, 75).	Human causality and therapeutic selectivity have not been established.
Diabetic osteoporosis	Osteoblasts, osteocytes, and BMSCs	Level B	FtMt-, METTL3-, Nrf2-, AMPK-, and glucolipotoxicity-related studies support ferroptotic involvement across several osteoblast, osteocyte, and BMSC models (8, 19, 21, 66, 67, 81, 104).	Most studies are single-centre animal investigations with limited cell-specific genetic validation.
Glucocorticoid-induced osteoporosis	BMSCs, osteoblasts, and osteocytes	Level A	PI3K/AKT/mTOR and Parkin/ACSL4 studies support BMSC injury; exosome studies support osteoblast protection; GPX4 deletion, specific rescue, and PSMD14/SLC7A11 intervention establish osteocyte involvement (25, 79, 82, 90, 106, 109).	Evidence is preclinical and heterogeneous across cells, models, and delivery systems; human efficacy is unknown.
Ageing-related osteoporosis	Osteoblasts, osteocytes, and BMSCs	Level A	VDR/Nrf2/GPX4 in accelerated-ageing osteoblasts, ATF3/TFR1 intervention in naturally aged osteocytes, and Steap4 lineage deletion/rescue in senescent BMSCs provide complementary *in vivo* evidence (68, 89, 105).	Evidence is confined to animal/cell models and does not establish a validated mechanism in natural human ageing.
Iron-overload-related osteoporosis	Osteoblasts and differentiating osteoclasts	Level A	Direct osteoblast models link iron burden to NOX4-driven ferroptosis and rescue; Wnt activation provides an independent intervention, while artesunate provides a pharmacological osteoclast-induction model (18, 87, 102).	Human studies remain associative; the osteoclast study does not prove that iron overload alone invariably causes mature-osteoclast ferroptosis.
Inflammatory bone loss	Osteoclasts and osteoblasts	Level C	ROS and RANKL mechanisms are well supported, but most studies lack evidence of iron dependence and ferroptosis-specific rescue (31, 32, 97, 98, 125, 128, 129).	General inflammatory oxidative stress should not be equated with ferroptosis.

## 7 Therapeutic strategies targeting ferroptosis in osteoporosis

Ferroptosis-targeted treatment of osteoporosis must be designed according to bone-cell identity, disease subtype, and turnover state. The principal experimental contexts are iron-overload bone loss for iron chelation, radical-trapping rescue, and pharmacological osteoclast induction; diabetic bone injury for Nrf2- or metabolism-directed protection; glucocorticoid injury for BMSC, osteoblast, and osteocyte protection; and ageing models for VDR-, ATF3/TFR1-, or Steap4-directed approaches (18, 19, 21, 25, 67, 68, 75, 79, 81, 82, 87, 89, 90, 101, 102, 104–106, 109). HIF-1α/NCOA4 modulation, MZF1-associated regulation, artesunate, and zoledronate-related findings suggest candidate osteoclast directions, but none has established cell-selective clinical benefit in osteoporosis (34, 38, 101, 102). The therapeutic objective is selective restoration of iron and lipid-redox homeostasis rather than uniform inhibition or induction of ferroptosis (**Figure 6**). Representative ferroptosis-targeted therapeutic strategies are summarised in **Table 5**.

**Figure 6:**
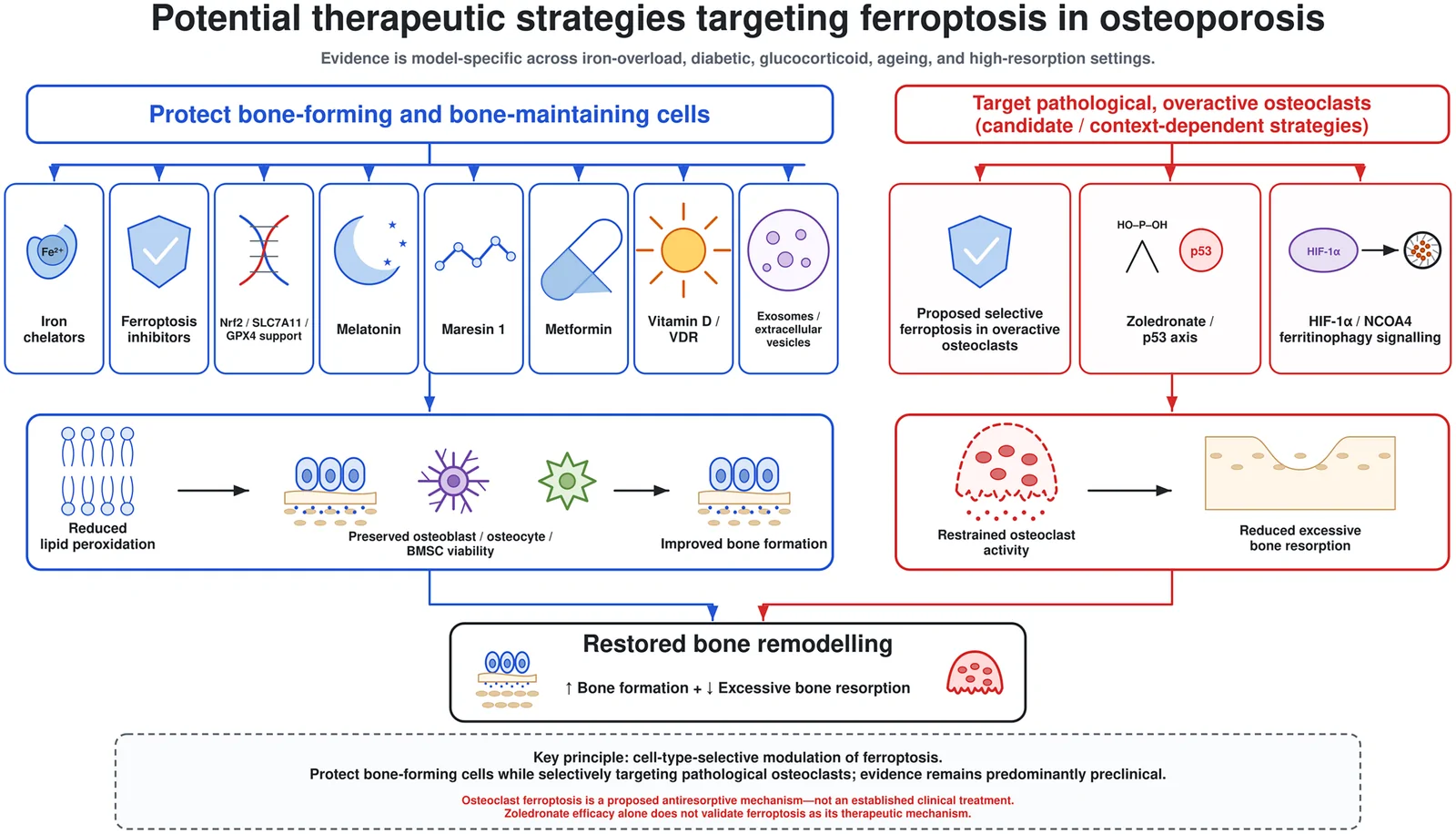
Potential therapeutic strategies targeting ferroptosis in osteoporosis. Cell colours follow **Figure 4**. Osteoclast ferroptosis is a proposed selective antiresorptive mechanism, not an established clinical treatment. Clinical efficacy of zoledronate does not by itself validate ferroptosis as its therapeutic mechanism.

**Table 5:** Representative ferroptosis-targeted therapeutic strategies in osteoporosis.

Representative agent/strategy	Major target/pathway	Main protected or targeted cell type(s)	Principal effect on ferroptosis and bone remodelling
A. Bone-forming/bone-maintaining cell-protective anti-ferroptotic strategies			
Deferoxamine (DFO)	Iron chelation; ↓ labile Fe²^+^, Fenton chemistry, ROS, and lipid peroxidation (8, 18, 90, 105).	Osteoblasts, osteocytes, and BMSCs	Mechanistic rescue in iron-overload, glucocorticoid-osteocyte, ageing-BMSC, diabetic, and tobacco-toxin models; systemic iron status and toxicity require monitoring.
Ferrostatin-1/Liproxstatin-1	Radical-trapping anti-ferroptosis; inhibits phospholipid peroxidation (17, 18, 20, 21, 25, 35, 36).	Osteoblasts, osteocytes, and BMSCs	Preserves cell viability and reduces ferroptotic injury. Model context: Postmenopausal, diabetic, glucocorticoid and iron-overload models.
Metabolic/proteostasis restoration (melatonin, PSMD14 activation, or Parkin restoration)	PI3K/AKT/mTOR; PSMD14-dependent SLC7A11 stabilisation; Parkin-dependent ACSL4 degradation (25, 90, 106).	BMSCs, osteocytes, or osteoblasts, depending on the model	Supports cell-specific protection in glucocorticoid and diabetic models; PSMD14 and bone-targeted Parkin delivery remain preclinical.
Metformin	AMPK/Nrf2 signalling; antioxidant and metabolic protection (81).	Osteoblasts	Reduces ferroptosis sensitivity and improves osteogenic function. Model context: Diabetic models.
Vitamin D/VDR	VDR-associated redox homeostasis; support of antioxidant defences (68).	Osteoblasts	Protects bone-forming cells and favours osteogenic differentiation. Model context: Ageing-related models.
Maresin 1	Nrf2/SLC7A11/GPX4-associated lipid-peroxide defence (19).	Osteoblasts	Preserves osteogenic cell viability and supports bone formation. Model context: Diabetic high-glucose models.
Exosomes/extracellular vesicles	Study-specific mechanisms: ferritinophagy inhibition in glucocorticoid-exposed osteoblasts (79); redox/SLC7A11/GPX4-associated improvement with incomplete death-mechanism validation (82); PI3K/AKT-associated BMSC protection (109); targeted curcumin delivery to BMSCs (108); miR-335-3p/PTGS2 regulation in high-glucose osteoblast culture (133).	Osteoblasts and BMSCs, depending on the preparation and model	Preserves osteogenic function in glucocorticoid, smoking-related, or diabetic experimental contexts. Donor source, cargo, route, and mechanistic certainty are not interchangeable.
B. Osteoclast-targeting/context-dependent candidate strategies			
Zoledronate	Osteoclast ferroptosis through an FBXO9/p53-related mechanism (38).	Overactive osteoclasts	Mechanistic evidence arises from a bisphosphonate-related jaw-osteonecrosis context. Relevance to high-resorption osteoporosis is proposed, not an established selective clinical mechanism.
HIF-1α/NCOA4, MZF1/Nrf2/GPX4, or artesunate modulation	Context-dependent ferritinophagy, antioxidant defence, or pharmacological ferroptosis induction (34, 101, 102).	Differentiating or overactive osteoclasts	Preclinical candidate mechanisms; MZF1 evidence lacks specific rescue, artesunate is not established therapy, and osteoclast-specific clinical efficacy is unproven.

BMSCs, bone marrow mesenchymal stem cells; DFO, deferoxamine; Fe, iron; ROS, reactive oxygen species; VDR, vitamin D receptor.

Most strategies listed are supported predominantly by preclinical evidence.

### 7.1 Iron homeostasis modulation and ferroptosis inhibitors

Iron chelation and radical-trapping ferroptosis inhibition represent the most direct experimental strategies (9, 18, 20, 21, 35, 36). Deferoxamine can reduce pathological iron accumulation and limit iron-catalysed Fenton chemistry, while ferrostatin-1 and liproxstatin-1 inhibit propagation of lipid radicals (9, 18, 35, 36). These agents have also served as mechanistic rescue tools in osteocyte and BMSC models, including glucocorticoid- and ageing-related studies (90, 105). They support causal interpretation in preclinical models but are not established osteoporosis treatments.

However, these interventions should be interpreted cautiously. Physiological iron availability supports osteoblast mineralisation and skeletal homeostasis, whereas severe iron deficiency can reduce both bone formation and resorption (11, 51). Therefore, iron chelation should be regarded as a strategy for restoring iron homeostasis rather than simply lowering iron levels. Similarly, ferroptosis inhibitors may protect osteoblasts, osteocytes, and BMSCs, but broad inhibition could preserve overactive osteoclasts or interfere with physiological redox signalling. Most evidence remains preclinical. Systemic safety cannot be inferred from bone-cell protection: deferoxamine-associated visual and auditory neurotoxicity has been documented in treated patients (134), while long-term human safety of ferrostatin-1 and liproxstatin-1 in osteoporosis remains unestablished.

Because GPX4 is a selenoprotein, selenium status may influence its catalytic capacity (24, 76). Selenium-deficient mice developed impaired bone microarchitecture (78), whereas a randomised trial in postmenopausal women did not show that short-term selenium supplementation improved bone-turnover markers (77). Selenium should therefore be evaluated only as a possible conditional modifier of GPX4 function and should not be presented alongside iron chelators or ferroptosis inhibitors as an established osteoporosis therapy.

### 7.2 Metabolic and antioxidant modulators

Several metabolic or antioxidant agents may protect bone-forming cells by restoring anti-ferroptosis defence systems. Melatonin has been reported to suppress ferroptosis in BMSCs under glucocorticoid stress by activating the PI3K/AKT/mTOR pathway. In diabetic osteoporosis, melatonin may also protect osteoblasts through Nrf2/HO-1-related antioxidant signalling (25, 67). These findings indicate that melatonin may act not only as a general antioxidant but also as a regulator of ferroptosis-sensitive survival pathways in osteoblast-lineage cells (25, 67).

Maresin 1, metformin, and vitamin D/VDR activation provide additional examples of metabolic or antioxidant protection (19, 68, 81). More recent proteostasis-directed candidates include activation of PSMD14 to stabilise SLC7A11 in glucocorticoid-exposed osteocytes and bone-targeted Parkin mRNA delivery to promote ACSL4 degradation in glucocorticoid-exposed BMSCs (90, 106). These interventions improved skeletal outcomes in preclinical models, but their delivery systems, off-target effects, and clinical safety require independent validation.

### 7.3 Exosomes and extracellular vesicles

Exosome and extracellular-vesicle interventions should be distinguished by donor source, recipient cell, cargo, route, and disease model. Vascular endothelial cell-derived exosomes protected glucocorticoid-exposed osteoblasts through suppression of ferritinophagy-dependent ferroptosis and were administered intravenously in the associated *in vivo* model; no causal vesicular cargo was identified (79). In a separate dexamethasone model, mouse bone-marrow endothelial progenitor cell-derived vesicles (50 µg; approximately 3.45 × 10¹^0^ vesicles) were injected through the tail vein once weekly for four weeks and improved bone microarchitecture together with osteoblastic redox/SLC7A11/GPX4-associated readouts, but did not fully establish the death mechanism or identify a causal vesicular cargo (82). Human umbilical-cord mesenchymal stromal cell-derived exosomes (100 µg) were administered intramuscularly every two days for eight weeks in a glucocorticoid-induced mouse model and promoted human BMSC proliferation and osteogenesis in corresponding cell experiments through PI3K/AKT-associated suppression of lipid peroxidation (109). A recipient-cell signalling change should not be described as a delivered RNA or protein unless that cargo has been directly identified.

Engineered vesicles provide a more defined delivery example. Fut6-overexpressing HEK293T-cell exosomes were modified with the bone-targeting peptide (AspSerSer)6 and loaded with curcumin; intravenous tail-vein administration was directly evaluated for biodistribution, and the formulation preserved BMSC osteogenic potential and mitigated bone loss in a smoking-related mouse model (108). The biodistribution experiment supports systemic bone targeting, but should not be used alone to infer every detail of the therapeutic dosing schedule. More recently, bEND.3 endothelial-cell exosomes were linked to miR-335-3p-dependent regulation of PTGS2 in high-glucose-exposed MC3T3-E1 osteoblasts; miRNA inhibition and a reporter assay supported the cargo–target relationship (133). The cell-culture treatment and associated human serum measurements do not establish human therapeutic efficacy or a validated *in vivo* delivery route. Source heterogeneity, cargo characterisation, dose standardisation, targeting efficiency, storage stability, and safety remain barriers to translation.

### 7.4 Epigenetic restoration of GPX4

Epigenetic regulation provides a more specific therapeutic direction. DNMT-mediated hypermethylation of the GPX4 promoter can suppress GPX4 transcription, weaken lipid-peroxide detoxification, and promote osteoblast ferroptosis (75). DNMT inhibition may restore GPX4 expression and reduce ferroptotic bone loss, suggesting that epigenetic therapy may protect osteoblast-lineage cells by reactivating endogenous anti-ferroptosis defences (75).

This strategy is mechanistically attractive because it targets upstream suppression of GPX4 rather than merely clearing ROS after injury has occurred. Nevertheless, broad DNMT inhibition may affect many genes other than GPX4 and may produce cell-type-independent effects (75). Future work should determine whether GPX4 restoration can be achieved more selectively in osteoblast-lineage cells, for example through bone-targeted delivery systems or more precise epigenetic modulators.

### 7.5 Osteoclast-targeted induction of ferroptosis

Ferroptosis should not always be inhibited in osteoporosis: under high-resorption conditions, selective induction of osteoclast ferroptosis could reduce excessive bone degradation (34, 38, 102). Zoledronic acid promoted osteoclast ferroptosis through an FBXO9/p53-related mechanism in a jaw-osteonecrosis study (38), whereas artesunate induced lipid-peroxidation and ferroptotic mitochondrial features in differentiating osteoclasts and reduced iron-overload-associated bone loss (102). MZF1 loss produced the opposite remodelling direction—less iron/lipid-oxidation signalling with greater osteoclastogenesis—but its ferroptosis interpretation lacked specific rescue (101). These are mechanistic preclinical observations, not evidence of established selective therapy.

However, osteoclast-targeted induction of ferroptosis must be highly selective. Nonspecific enhancement of oxidative stress may injure osteoblasts, osteocytes, BMSCs, and the bone marrow microenvironment. Future strategies should therefore increase ferroptotic vulnerability specifically in overactive osteoclasts rather than induce generalised oxidative injury throughout bone (17, 18, 20, 21, 25, 34, 38). Potential approaches include osteoclast-targeted delivery or modulation of validated downstream components, but an optimal therapeutic window and long-term preservation of remodelling coupling remain unestablished.

### 7.6 Cell-type-specific therapeutic logic

The central therapeutic challenge is cell-type specificity. Anti-ferroptotic strategies can preserve osteoblasts, osteocytes, and BMSCs, whereas excessive protection may prolong osteoclast survival in high-resorption states (17, 18, 20, 21, 25, 34, 38). The same intervention may therefore have opposite consequences depending on the dominant pathological cell population.

A practical framework should distinguish protective anti-ferroptosis in low-bone-formation states from selective ferroptosis induction in overactive osteoclasts. Iron chelation, radical trapping, metabolic regulation, extracellular-vesicle delivery, epigenetic restoration of GPX4, and osteoclast-directed approaches are all candidates, but translation depends on targeting, safety, and disease classification.

### 7.7 Translational evidence thresholds and preclinical evaluation framework

Before a ferroptosis-targeted intervention advances into translational osteoporosis research, it should meet at least four preclinical thresholds. First, iron-dependent membrane phospholipid peroxidation, rather than nonspecific reactive oxygen species injury, should be demonstrated in a defined bone-cell population. Second, genetic or pharmacological rescue should establish a causal relationship between the mechanism and bone-formation or bone-resorption outcomes. Third, the intervention should improve bone microarchitecture, bone strength, or bone-turnover indices *in vivo*. Fourth, it should be shown not to cause anaemia, organ iron accumulation, or injury to non-target bone cells through systemic disruption of iron homeostasis or redox defence. At the cellular level, validation should pair death and membrane-peroxidation measurements with iron chelation and a lipid-radical-trapping rescue, and preferably with cell-specific GPX4/SLC7A11 manipulation or validation of an alternative defence system (15, 16, 18, 21, 35, 36, 75). Functional endpoints should include mineralisation for osteoblasts/BMSCs, resorption-pit activity for osteoclasts, and RANKL/OPG-linked co-culture effects for osteocytes. *In vivo* evaluation should combine micro-CT with dynamic histomorphometry, formation/resorption indices, and mechanical strength or fracture-related outcomes; improved BMD alone does not identify the cellular mechanism (17, 18, 21, 75).

No biomarker or ferroptosis signature discussed here has been validated to diagnose ferroptosis specifically in human bone or to select patients who will benefit from ferroptosis-targeted therapy. Ferritin is influenced by systemic iron stores and inflammation; MDA/4-HNE and altered GPX4 expression are not specific to ferroptotic death. Exploratory serum miR-335-3p/PTGS2 associations in diabetic osteoporosis illustrate biomarker discovery, not validation of bone-localised ferroptosis or prediction of treatment response (133). A candidate panel could combine iron indices, lipid-oxidation products, bone-turnover markers and imaging, but would need independent validation and prospective testing within each osteoporosis subtype. No clinical trial included in this review establishes fracture reduction, improved BMD or bone-turnover benefit attributable specifically to ferroptosis targeting. The anti-fracture efficacy of established osteoporosis drugs cannot be used as evidence for that unproven mechanism (135–140).

## 8 Discussion and perspectives

Ferroptosis links endocrine and metabolic stress, membrane lipid peroxidation, and imbalanced bone remodelling. Postmenopausal status, diabetes, glucocorticoids, ageing, and iron overload enter this process through distinct upstream mechanisms, and multiple models now demonstrate iron dependence, membrane lipid peroxidation, specific rescue, or genetic intervention (17, 18, 20, 21, 25, 104).

Its skeletal effect is cell-type dependent. Ferroptosis generally impairs bone formation and remodelling communication in osteoblasts, osteocytes, and BMSCs, but may restrict resorption when selectively induced in overactive osteoclasts (17, 18, 20, 21, 25, 34, 38). Interventions should therefore be aligned with cell identity, disease subtype, and bone-turnover status.

The dominant targets also differ among osteoporosis subtypes, precluding a uniform therapeutic approach. Newer primary studies strengthen natural-ageing osteocyte and BMSC evidence, glucocorticoid osteocyte and BMSC mechanisms, and pharmacological osteoclast induction, but all remain preclinical (89, 90, 101, 102, 105, 106). Established anti-osteoporosis drugs have proven anti-fracture efficacy (135–140). Ferroptosis has not been established as their principal clinical mechanism.

Future work should combine cell-specific models, lineage tracing, single-cell and spatial analyses, iron and lipid profiling, and criteria that integrate iron dependence, membrane phospholipid peroxidation, GPX4 or alternative defence-system function, and ferroptosis-specific rescue (141). In bone tissue, these considerations are particularly important because whole-bone assays mix several cell populations and transient death events may be missed; moreover, sample handling and assay suitability require explicit validation in mineralised tissue. ROS probes, lipid-oxidation measurements, iron assays, oxidised-phospholipid profiling, spatial readouts, and functional rescue should therefore be interpreted together rather than treating a single stain or expression change as diagnostic (141, 142). Ferroptosis should also be distinguished from caspase-associated apoptosis and RIPK3–MLKL-mediated necroptosis through parallel pathway assays and selective rescue, because death mechanisms can coexist (15, 16, 27, 28, 143). Autophagy can facilitate ferroptosis through NCOA4-dependent ferritin degradation, but increased autophagic flux alone neither establishes ferroptosis nor proves autophagy-dependent death (41, 42, 54). Bone- or cell-targeted delivery must ultimately be evaluated against skeletal benefit, systemic safety, disease stage, and the intervention window.
