# Supplementary material for: Ferroptosis in osteoporosis: core mechanisms, cell-specific effects in bone remodelling, and therapeutic perspectives
Source: Front Endocrinol (Lausanne). 2026 Sep 25;17:1945454. doi: 10.3389/fendo.2026.1945454 (PMC13650743; doi:10.3389/fendo.2026.1945454)
Supplement: Supplementary file 1 [file Table1.docx]

**Supplementary Table 1**

This table records the reproducible searches actually executed during revision. All searches were run on 06 September 2026 and used a publication-date end point of the same date. PubMed and Web of Science use their database-specific date fields. Counts are the database-displayed hits at execution, before deduplication or manual assessment.

PubMed

| Search purpose | Complete Boolean search string | Actual execution date | Records retrieved |
| --- | --- | --- | --- |
| Bone-cell and osteoporosis evidence | (ferroptosis[Title/Abstract] OR ferroptotic[Title/Abstract]) AND (osteoporosis[Title/Abstract] OR osteoporotic[Title/Abstract] OR "bone loss"[Title/Abstract] OR "bone remodelling"[Title/Abstract] OR "bone remodeling"[Title/Abstract] OR osteoblast*[Title/Abstract] OR osteocyte*[Title/Abstract] OR osteoclast*[Title/Abstract] OR "bone marrow mesenchymal stem cell"[Title/Abstract] OR "bone marrow mesenchymal stem cells"[Title/Abstract] OR "bone marrow mesenchymal stromal cell"[Title/Abstract] OR "bone marrow mesenchymal stromal cells"[Title/Abstract] OR BMSC*[Title/Abstract]) AND ("1900/01/01"[Date - Publication] : "2026/09/06"[Date - Publication]) AND english[Language] | 2026-09-06 | n = 532 |
| Osteoporosis-subtype evidence | (ferroptosis[Title/Abstract] OR ferroptotic[Title/Abstract]) AND (osteoporosis[Title/Abstract] OR osteoporotic[Title/Abstract] OR "bone loss"[Title/Abstract] OR "bone remodelling"[Title/Abstract] OR "bone remodeling"[Title/Abstract] OR osteoblast*[Title/Abstract] OR osteocyte*[Title/Abstract] OR osteoclast*[Title/Abstract] OR "bone marrow mesenchymal stem cell"[Title/Abstract] OR "bone marrow mesenchymal stem cells"[Title/Abstract] OR "bone marrow mesenchymal stromal cell"[Title/Abstract] OR "bone marrow mesenchymal stromal cells"[Title/Abstract] OR BMSC*[Title/Abstract]) AND (postmenopausal[Title/Abstract] OR "oestrogen deficiency"[Title/Abstract] OR "estrogen deficiency"[Title/Abstract] OR diabetic[Title/Abstract] OR diabetes[Title/Abstract] OR hyperglyc*[Title/Abstract] OR glucolipotoxic*[Title/Abstract] OR glucocorticoid*[Title/Abstract] OR dexamethasone[Title/Abstract] OR ageing[Title/Abstract] OR aging[Title/Abstract] OR senescence[Title/Abstract] OR smoking[Title/Abstract] OR "cigarette smoke"[Title/Abstract] OR "iron overload"[Title/Abstract] OR inflammatory[Title/Abstract] OR inflammation[Title/Abstract]) AND ("1900/01/01"[Date - Publication] : "2026/09/06"[Date - Publication]) AND english[Language] | 2026-09-06 | n = 322 |
| Therapeutic/intervention evidence | (ferroptosis[Title/Abstract] OR ferroptotic[Title/Abstract]) AND (osteoporosis[Title/Abstract] OR osteoporotic[Title/Abstract] OR "bone loss"[Title/Abstract] OR "bone remodelling"[Title/Abstract] OR "bone remodeling"[Title/Abstract] OR osteoblast*[Title/Abstract] OR osteocyte*[Title/Abstract] OR osteoclast*[Title/Abstract] OR "bone marrow mesenchymal stem cell"[Title/Abstract] OR "bone marrow mesenchymal stem cells"[Title/Abstract] OR "bone marrow mesenchymal stromal cell"[Title/Abstract] OR "bone marrow mesenchymal stromal cells"[Title/Abstract] OR BMSC*[Title/Abstract]) AND (therap*[Title/Abstract] OR treatment*[Title/Abstract] OR ferrostatin*[Title/Abstract] OR liproxstatin*[Title/Abstract] OR deferoxamine[Title/Abstract] OR "iron chelation"[Title/Abstract] OR chelator*[Title/Abstract] OR melatonin[Title/Abstract] OR metformin[Title/Abstract] OR exosome*[Title/Abstract] OR "extracellular vesicle"[Title/Abstract] OR "extracellular vesicles"[Title/Abstract] OR zoledron*[Title/Abstract] OR Nrf2[Title/Abstract] OR "Wnt/beta-catenin"[Title/Abstract] OR "Wnt/β-catenin"[Title/Abstract]) AND ("1900/01/01"[Date - Publication] : "2026/09/06"[Date - Publication]) AND english[Language] | 2026-09-06 | n = 468 |
| Core mechanistic background | (ferroptosis[Title/Abstract] OR ferroptotic[Title/Abstract]) AND ("iron homeostasis"[Title/Abstract] OR "iron metabolism"[Title/Abstract] OR "lipid peroxidation"[Title/Abstract] OR GPX4[Title/Abstract] OR SLC7A11[Title/Abstract] OR ferritinophagy[Title/Abstract] OR mitophagy[Title/Abstract] OR FSP1[Title/Abstract] OR DHODH[Title/Abstract] OR GCH1[Title/Abstract] OR NOX4[Title/Abstract] OR DNMT*[Title/Abstract] OR NCOA4[Title/Abstract] OR ACSL4[Title/Abstract] OR LPCAT3[Title/Abstract]) AND ("1900/01/01"[Date - Publication] : "2026/09/06"[Date - Publication]) AND english[Language] | 2026-09-06 | n = 16,694 |
| GPX4/selenium background | GPX4[Title/Abstract] AND (selenium[Title/Abstract] OR selenoprotein*[Title/Abstract] OR selenocysteine[Title/Abstract]) AND ("1900/01/01"[Date - Publication] : "2026/09/06"[Date - Publication]) AND english[Language] | 2026-09-06 | n = 603 |

Web of Science Core Collection

| Search purpose | Complete Boolean search string | Actual execution date | Records retrieved |
| --- | --- | --- | --- |
| Bone-cell and osteoporosis evidence | TS=((ferroptosis OR ferroptotic) AND (osteoporosis OR osteoporotic OR "bone loss" OR "bone remodelling" OR "bone remodeling" OR osteoblast* OR osteocyte* OR osteoclast* OR "bone marrow mesenchymal stem cell" OR "bone marrow mesenchymal stem cells" OR "bone marrow mesenchymal stromal cell" OR "bone marrow mesenchymal stromal cells" OR BMSC*)) AND LA=(English) AND DOP=1900-01-01/2026-09-06 | 2026-09-06 | n = 579 |
| Osteoporosis-subtype evidence | TS=((ferroptosis OR ferroptotic) AND (osteoporosis OR osteoporotic OR "bone loss" OR "bone remodelling" OR "bone remodeling" OR osteoblast* OR osteocyte* OR osteoclast* OR "bone marrow mesenchymal stem cell" OR "bone marrow mesenchymal stem cells" OR "bone marrow mesenchymal stromal cell" OR "bone marrow mesenchymal stromal cells" OR BMSC*) AND (postmenopausal OR "oestrogen deficiency" OR "estrogen deficiency" OR diabetic OR diabetes OR hyperglyc* OR glucolipotoxic* OR glucocorticoid* OR dexamethasone OR ageing OR aging OR senescence OR smoking OR "cigarette smoke" OR "iron overload" OR inflammatory OR inflammation)) AND LA=(English) AND DOP=1900-01-01/2026-09-06 | 2026-09-06 | n = 368 |
| Therapeutic/intervention evidence | TS=((ferroptosis OR ferroptotic) AND (osteoporosis OR osteoporotic OR "bone loss" OR "bone remodelling" OR "bone remodeling" OR osteoblast* OR osteocyte* OR osteoclast* OR "bone marrow mesenchymal stem cell" OR "bone marrow mesenchymal stem cells" OR "bone marrow mesenchymal stromal cell" OR "bone marrow mesenchymal stromal cells" OR BMSC*) AND (therap* OR treatment* OR ferrostatin* OR liproxstatin* OR deferoxamine OR "iron chelation" OR chelator* OR melatonin OR metformin OR exosome* OR "extracellular vesicle" OR "extracellular vesicles" OR zoledron* OR Nrf2 OR "Wnt/beta-catenin" OR "Wnt/β-catenin")) AND LA=(English) AND DOP=1900-01-01/2026-09-06 | 2026-09-06 | n = 503 |
| Core mechanistic background | TS=((ferroptosis OR ferroptotic) AND ("iron homeostasis" OR "iron metabolism" OR "lipid peroxidation" OR GPX4 OR SLC7A11 OR ferritinophagy OR mitophagy OR FSP1 OR DHODH OR GCH1 OR NOX4 OR DNMT* OR NCOA4 OR ACSL4 OR LPCAT3)) AND LA=(English) AND DOP=1900-01-01/2026-09-06 | 2026-09-06 | n = 17,849 |
| GPX4/selenium background | TS=(GPX4 AND (selenium OR selenoprotein* OR selenocysteine)) AND LA=(English) AND DOP=1900-01-01/2026-09-06 | 2026-09-06 | n = 651 |
